# Predicting of elderly population structure and density by a novel grey fractional-order model with theta residual optimization: a case study of Shanghai City, China

**DOI:** 10.1186/s12877-023-04197-2

**Published:** 2023-09-16

**Authors:** Xiaojun Guo, Jiaxin Li, Xinyao Zhu, Yingjie Yang, Jingliang Jin

**Affiliations:** 1 School of Science, Nantong University, Nantong, 226019 China; 2 Institute of Artificial Intelligence, De Montfort University, Leicester, LE1 9BH UK

**Keywords:** Population aging, Elderly population prediction, Grey prediction model, Fractional order accumulation, Theta residual optimization

## Abstract

**Background:**

Accurately predicting the future development trend of population aging is conducive to accelerating the development of the elderly care industry. This study constructed a combined optimization grey prediction model to predict the structure and density of elderly population.

**Methods:**

In this paper, a GT-FGM model is proposed, which combines Theta residual optimization with fractional-order accumulation operator. Fractional-order accumulation can effectively weaken the randomness of the original data sequence. Meanwhile, Theta residual optimization can adjust parameter by minimizing the mean absolute error. And the population statistics of Shanghai city from 2006 to 2020 were selected for prediction analysis. By comparing with the other traditional grey prediction methods, three representative error indexes (MAE, MAPE, RMSE) were conducting for error analysis.

**Results:**

Compared with the FGM model, GM (1,1) model, Verhulst model, Logistic model, SES and other classical prediction methods, the GT-FGM model shows significant forecasting advantages, and its multi-step rolling prediction accuracy is superior to other prediction methods. The results show that the elderly population density in nine districts in Shanghai will exceed 0.5 by 2030, among which Huangpu District has the highest elderly population density, reaching 0.6825. There has been a steady increase in the elderly population over the age of 60.

**Conclusions:**

The GT-FGM model can improve the prediction accuracy effectively. The elderly population in Shanghai shows a steady growth trend on the whole, and the differences between districts are obvious. The government should build a modern pension industry system according to the aging degree of the population in each region, and promote the balanced development of each region.

## Introduction

With the rapid economic development, China's population aging problem has become increasingly prominent. Since China entered an aging society in 1999, the trend of population aging has become more and more significant, which has seriously affected economic development. According to the "Research Report on Predicting Development Trend of China's Population Aging" released in 2006, China will become an irreversible aging society after entering the twenty-first century. Population aging is an important social problem that China is about to face. With the continuous improvement of people's quality of life and medical level, China's population aging problem is becoming more and more serious. China's aging process presents a development trend of large scale, accelerated growth rate, long duration and regional imbalance. Under the background of an aging population, the elderly care industry is developing rapidly, and the scale of social elderly care institutions is also expanding, but it still cannot meet the needs of the elderly population for elderly care institutions. Unbalanced development among regions and uneven distribution of pension infrastructure resources further lead to the spatial imbalance in the distribution of the elderly population. The scientific and reasonable prediction of the development trend of population aging is helpful to provide data and theoretical support for the government to formulate relevant population policies. Predicting the number and density distribution of the elderly population can optimize the allocation of endowment resources, promote balanced development among regions, so as to meet the needs of the elderly population for the elderly and reduce the burden of society. Accurately predicting the structure of the elderly population can better cope with the increasing pressure of the elderly care, and is conducive to promoting the development of the elderly care industry, improving the elderly care service system and improving the infrastructure construction.

### Influential factors of aging population

China's aging population is an increasingly serious social problem, which is caused by many factors. Here are some of the main influencing factors:

1. Birth policy. China's family planning policy has been in place since the 1970s to control population growth. While the policy achieved its goals to some extent, it also led to a dramatic drop in fertility. According to China's National Bureau of Statistics, the total fertility rate in 2019 was 1.69, meaning that each woman had fewer than two children on average. At the same time, with the development of medical technology, people are living longer, leading to an increase in the number of elderly people.
2. Economic development. With the rapid economic development, China's standard of living and social security have been significantly improved. However, at the same time, the cost of living is also rising, which puts increasing pressure on family finances. Many young people believe that giving birth will put too much financial burden on their families, so they are more inclined to choose personal development and career pursuits. In addition, with the improvement of education, more and more women are choosing to pursue career development instead of just focusing on family and children.
3. Urban and rural differences. There are big differences between urban and rural areas in China, as well as different levels of aging. The aging rate is higher in cities than in rural areas because urban families are under greater economic pressure and so tend to have fewer children. Fertility rates are generally higher in rural areas, where the financial burden on families is relatively low and where the traditional belief that having more children increases the family labor force exists.
4. Medical treatment level. As medical technology continues to improve, people are living longer, leading to an increase in the number of elderly people. The growing number of elderly people not only increases the burden of old-age security, but also brings more challenges to medical and health services, as the elderly are more prone to chronic diseases and need more medical and health service support.

The combined effect of these factors leads to the current situation of China's aging population. The aging trend will bring challenges and opportunities to China's economy, society, politics and other aspects, so it is necessary to take corresponding measures to cope with the impact of aging.

### Literature review of prediction models

 Population prediction takes the law of population development as the main body to determine the parameters, and the acquisition of relevant data and the selection of prediction algorithm greatly affect the accuracy of prediction results. The existing models used in population prediction research mainly include linear regression model [1], Malthus model [2], Logistic model [3], BP neural network model [4] and Grey prediction model [5]. Linear regression model requires population data to change smoothly with obvious linear trend, which is suitable for modeling the relationship between continuous dependent variable and one or more continuous independent variables. Its limitation is that it assumes that the relationship between dependent variable and independent variable is linear, but with the development of economic society, population is difficult to change linearly. Malthus model is a common model of population growth, which assumes exponential growth of population and linear growth of resources. The model is suitable for studying the long-term trend of population growth, but its limitation is that it ignores other factors in the real world, such as technological progress and social policies. Logistic model is suitable for building a model of classification problem. It can divide the population into two or more categories, such as male and female, old and young, etc. Its limitation is that it assumes that the classification decision boundary is linear, so if the data does not conform to this assumption, the accuracy of the model will be affected. BP neural network model is an artificial neural network model, which can deal with nonlinear relations and is suitable for complex problems in population prediction. It can be trained to automatically learn features and predict future population trends. The limitation of this model is that it requires a large amount of training data and is prone to overfitting problems. Grey prediction model is suitable for the prediction of small samples, nonlinear and uncertain problems. It establishes the model through the grey system analysis of the data [6–8]. However, the model requires data preprocessing, and the prediction results may be affected by the selection of model parameters and data quality.

Grey prediction is a method to predict the system with uncertain factors. It builds mathematical model through "small sample and poor information". Based on the improvement of the traditional grey model, the application range of the grey prediction model is widened. As the core model of grey prediction theory, GM(1,1) model is the most widely used [9]. However, the traditional GM(1,1) model is only applicable to the situation of exponential growth of time series and has certain limitations. Many scholars have improved the traditional grey model, and the new models have higher prediction accuracy. Those models are mainly optimized from several aspects: accumulation generation mode, initial value optimization, background value optimization and parameter estimation method. In the process of continuous development, grey system theory has gradually formed a theoretical system centered on grey prediction model, which can be divided into continuous type and discrete type [10], integer order and fractional order [11], linear and nonlinear [12], equal spacing and non-equal spacing [13], etc. In order to reduce the prediction error, Xie et al. built a new discrete grey prediction model (DGM) on the basis of AGO transformation, which can effectively overcome the problem of prediction accuracy [14]. The discrete DGM(1,1) model is suitable for predicting small and medium-sized data with discrete characteristics. It has low requirements on the continuity and integrity of data, and has good adaptability to nonlinear and large data differences. Wang introduced nonlinear parameters into the GMC(1,N) model, and obtained an improved grey prediction model (NGMC(1,n)) by means of convolution integral [15]. NGMC(1,n) model can solve the problems of nonlinear, non-periodic, non-stationarity and system change, and is suitable for stock price forecasting, seasonal sales forecasting, traffic forecasting and other fields. Hu et al. established a new non-equidistant grey prediction model (INEGM(1,1,t(2))) and optimized the background value [16]. INEGM(1,1,t(2)) model is applicable to many fields with seasonal and cyclical changes. Common applications include: flight passenger flow forecast, tourist flow forecast, agricultural product price forecast, power consumption forecast, etc. Wu et al. extended the traditional grey prediction model from integer order to fractional order and proposed a fractional order grey prediction model, which improved the prediction accuracy [17]. In this paper, fractional cumulative grey prediction model is adopted as a basic forecasting tool for population aging. The reason is that fractional cumulative model can effectively weaken the randomness of the original data series, and smooth the data series. It can be applied to population data with small sample, stable development trend and short forecasting cycle, which has certain advantages compared with other models. At the same time, fractional order accumulation can reduce the perturbation boundary of the prediction model, satisfy the new information priority principle, and improve the stability of prediction.

With the in-depth research on population aging, many scholars have begun to analyze the distribution of the aging population and the development trend of population aging. Developed countries have entered an aging society earlier than developing countries. European and American scholars have done relevant research on the problem of population aging. By collecting statistical data, Lindh et al. found that changes in the age structure of the population have a negative effect on economic development [18]. Tabata used the overlapping generation model to analyze the relationship between population aging and long-term economic growth rate, and found that population aging is not conducive to long-term economic growth [19]. Lutz et al. used the age structure of the actual population as a base to predict the total population and population growth rate in future years [20]. Grebenkov studied the abnormal growth regular of population aging, and found that the trend of population aging is becoming more and more severe, the number of elderly population has increased sharply, and the demand for elderly medical institutions is also increasing [21]. Hashimoto et al. established an iterative model to study the impact of medical demand brought by population aging on employment structure, and the research results show that population aging can promote the improvement of labor turnover rate [22]. Zhao et al. established a metabolism GM(1,1) model based on the traditional grey system theory, and tested the predictive performance of the model. The results show that the problem of population aging is still serious and needs active response [23]. Su et al. built a combined prediction model of population aging based on three single models: quadratic exponential smoothing prediction, modified grey prediction and BP neural network prediction. The prediction results show that the problem of population aging in China will become more and more serious in the future [24]. Sun constructed a combined prediction model for the prediction of the elderly population, and the prediction effect is better than other grey prediction models no matter in the sample or out of the sample [25]. Faced with the increasingly serious problem of population aging, all countries should take corresponding measures to reasonably predict the scale of the elderly population and the trend of population aging.

### Contribution and innovation

The grey model is a trend extrapolation model based on differential equations, which is mainly suitable for modeling small sample data. For data with nonlinear trends and long-term sequence, the prediction effect of the model is often not ideal, and the prediction error of the model can be adjusted by training samples. Theta predicting is a univariate predicting method that divides the raw data into several component patterns, called “ $\theta$ lines”, and obtains the final predicting results with a controllable curvature parameter [26]. Introducing the $\theta$ line in the grey prediction model to adjust the predicting error can improve the adaptability of the model. Theta prediction method, also known as exponential smoothing method with drift, corrects the local curvature of the time sequence based on the coefficient $\theta$. It can be directly applied to the second-order difference of the data sequence, which is beneficial to improve the prediction accuracy of the model [27]. On this basis, the original data sequence is decomposed into nonlinear trend items and linear trend items. The nonlinear trend item emphasizes the short-term characteristics of the data, and the linear trend item emphasizes the long-term characteristics of the data. Fractional accumulation can effectively weaken the randomness of the original data sequence. Considering that the prediction accuracy of the combined model is higher than that of a single model, the Theta residual optimization and the fractional accumulation grey prediction model are combined to improve the prediction accuracy of the model.

The grey prediction model is mainly aimed at the uncertain system of "small samples, poor information", and the population data series is in line with this feature. Therefore, the grey prediction model is combined with Theta residual optimization in this paper. The verification results of the model show that the new model has high prediction accuracy and generalization ability.

The contributions of this paper mainly include the following aspects:

1. The traditional grey prediction model is only applicable to the exponential growth of time series, and has certain limitations. It has become a research trend to improve the traditional grey prediction model. The GT-FGM model proposed in this paper uses fractional order accumulation to effectively weaken the randomness of the original data sequence, which satisfies the new information priority principle to a large extent. At the same time, particle swarm optimization algorithm is used to find the optimal order, so as to achieve better prediction effect. From the mechanism of the model, it makes up the defect of the traditional grey model.
2. Combining residual optimization of Grey Theta with fractional order accumulation model, a combinatorial optimization model is constructed. The Theta prediction model can adjust the local curvature of time series through parameters and minimize the average absolute error to adjust the advantages of parameters. The new model has better forecast robustness than the traditional grey prediction model. Meanwhile, compared with the single model, the prediction error of combinatorial optimization model is relatively small.
3. The prediction study on the number, density distribution and structure of the elderly population can fully explore the development trend of population aging and provide a reliable prediction method for the research in the field of gerontology. Demographic data, which can be much more informed by long-term time series, is susceptible to many factors. The longer the population time series, the lower its reliability, population data can not show an exponential growth trend. Therefore, the traditional grey model can not predict the population problem well, and the growth rate of the predicted data remains unchanged, which is inconsistent with the actual situation. Accordingly, for the population system, the new GT-FGM model has some advantages. It can mine the information of population data series well and adapt to the modeling of small data and uncertain system. Based on the residual optimization method, the heuristic algorithm is used to optimize the superparameters of the model. The proposed model has excellent adaptability, makes full use of the information value in the residual, and satisfies the new information priority principle.

### Organization and framework

The remainder of this paper is organized as follows. Section 2 provides an overview on traditional grey fractional order accumulation model, and introduces the detailed modeling process of and the Grey Theta fractional order accumulation grey model (GT-FGM) and its error analysis methods, and optimizes the hyperparameters using the particle swarm algorithm. Section 3 introduces the data sources of the total population and the elderly population in Shanghai, and gives the prediction results of the model. In Sect. 4, the GT-FGM model is adopted to predict the elderly population structure and density by comparing with other prediction methods. Finally, some conclusions and future work are drawn in Sect. 5.

## Methods

In this chapter, the definition of traditional fractional-order grey model is introduced, and then a Grey Theta fractional-order grey model (GT-FGM) based on residual optimization is established. Furthermore, the detailed modeling process and hyperparameter optimization method are given, and the innovation and improvement of the model are discussed.

### Basic model theory

The GM(1,1) model is the basic model of grey prediction theory and is widely used. The model builds a model through systematic behavioral data sequences, and can effectively predict and simulate data in the case of small samples. The GM(1,1) model is based on the grey system theory, through the continuous processing of discrete data, the differential equation is used to replace the difference equation. The new accumulated time sequence is used to replace the original time sequence, and then the differential equation is established [27]. The fractional-order GM(1,1) model (FGM(1,1)) converts the first-order cumulative generation in the traditional GM(1,1) model into fractional-order cumulative generation, and uses the fractional order to effectively weaken the randomness of the original data sequence. It can improve the prediction accuracy of the model and reduce the disturbance of the model solution.

The modeling process is as follows:

Step 1: Suppose the sequence of the original data is $X^{(0)} = \{ x^{(0)} (1),x^{(0)} (2), \cdots ,x^{(0)} (n)\}$, its corresponding r-order accumulation sequence is $X^{(r)} = \{ x^{(r)} (1),x^{(r)} (2), \cdots ,x^{(r)} (n)\}$,

1 $$x^{(r)} (k) = \sum\limits_{i = 1}^{k} {C_{k - i + r - 1}^{k - i} x^{(0)} (i)}$$

where stipulating $$C_{r - 1}^{0} = 1,C_{k}^{k + 1} = 0,C_{k - i + r - 1}^{k - i} = \frac{(k - i + r - 1)(k - i + r - 2) \cdots (r + 1)r}{{(k - i)!}}$$.

For the background value generation of $X^{(r)}$, a sequence close to mean generation is $Z^{(r)} = \{ z^{(r)} (1),z^{(r)} (2), \cdots ,z^{(r)} (n)\}$, where,

2 $$z^{(r)} (k) = \frac{1}{2}(x^{(r)} (k) + x^{(r)} (k - 1))$$

Step 2: The grey differential equation of r-order cumulative GM(1,1) model is

3 $$x^{(r)} (k) - x^{(r)} (k - 1) + az^{(r)} (k) = b$$

Its corresponding whitening differential equation can be expressed as

4 $$\frac{{{\text{d}}x^{(r)} (k)}}{{{\text{d}}t}} + ax^{(r)} (k) = b$$

where $a$ is the development coefficient and $b$ is the grey effect. The time response function can be obtained by solving the above differential equation

5 $$x^{(r)} (k + 1) = (x^{(0)} (1) - \frac{b}{a})e^{ - ak} + \frac{b}{a}$$

Step 3: Based on the principle of minimum sum of squares of errors, $$\left( {\begin{array}{*{20}c} {\hat{a}} \\ {\hat{b}} \\ \end{array} } \right)$$ can be calculated by the least square method as

6 $$\left[ {\begin{array}{*{20}c} {\hat{a}} \\ {\hat{b}} \\ \end{array} } \right] = (B^{{\text{T}}} B)^{ - 1} B^{{\text{T}}} Y$$

where,

7 $$B = \left[ {\begin{array}{*{20}c} { - z^{(r)} (2)} & 1 \\ { - z^{(r)} (3)} & 1 \\ \vdots & \vdots \\ { - z^{(r)} (n)} & 1 \\ \end{array} } \right],\,Y = \left[ {\begin{array}{*{20}c} {x^{(r)} (2) - x^{(r)} (1)} \\ {x^{(r)} (3) - x^{(r)} (2)} \\ \vdots \\ {x^{(r)} (n) - x^{(r)} (n - 1)} \\ \end{array} } \right]$$

Step 4: Substituting $\hat{a}$ and $\hat{b}$ into the equation, the time response function of the original sequence can be obtained as

8 $$\hat{x}^{(r)} (k + 1) = (x^{(0)} (1) - \frac{{\hat{b}}}{{\hat{a}}})e^{{ - \hat{a}k}} + \frac{{\hat{b}}}{{\hat{a}}},k = 1,2, \cdots ,n, \cdots$$

where $\hat{x}^{(r)} (k + 1)$ is the fitting value at the time of $k + 1$, and the sequence is

$$\hat{X}^{(r)} = \{ \hat{x}^{(r)} (1),\hat{x}^{(r)} (2), \cdots ,\hat{x}^{(r)} (n), \cdots \}$$

Step 5: The sequence $\hat{X}^{(r)} = \{ \hat{x}^{(r)} (1),\hat{x}^{(r)} (2), \cdots ,\hat{x}^{(r)} (n), \cdots \}$ is subjected to $r$-order subtractive reduction, then the fitting sequence of the original data is

9 $$\hat{X}^{(0)} = \alpha^{(r)} \hat{X}^{(r)} = \{ \alpha^{(1)} \hat{x}^{(r)(1 - r)} (1),\alpha^{(1)} \hat{x}^{(r)(1 - r)} (2), \cdots ,\alpha^{(1)} \hat{x}^{(r)(1 - r)} (n), \cdots \}$$

where,

10 $$\alpha^{(1)} \hat{x}^{(r)(1 - r)} (k) = \hat{x}^{(r)(1 - r)} (k) - \hat{x}^{(r)(1 - r)} (k - 1)$$

The fitting value of the original data sequence can be obtained as $\hat{x}^{(0)} (1),\hat{x}^{(0)} (2), \cdots ,\hat{x}^{(0)} (n)$, and the predicted value is $\hat{x}^{(0)} (n + 1),\hat{x}^{(0)} (n + 2), \cdots$.

### The modeling process of GT-FGM model

Grey model is mainly suitable for modeling small sample data, and it is based on the trend extrapolation principle of differential equation. However, when the data presents nonlinear relationship and large sample data, the prediction results of the model are often unsatisfactory, and the prediction error can be adjusted by training samples.

### Theta prediction skills

$Y_{t}$ is set as the observation sequence, and an $\theta$ line $Z_{t} (\theta )$ of coefficient $\theta$ can be obtained by the following formula

11 $$Z_{t}^{\prime \prime } (\theta ) = \theta Y^{\prime\prime}_{t} = \theta (Y_{t} - 2Y_{t - 1} + Y_{t - 2} ),t = 3, \cdots ,n$$

Its equivalent form is

12 $$Z_{t} (\theta ) = \theta Y_{t} + (1 - \theta )Z_{t} (0) = \theta Y_{t} + (1 - \theta )(A_{n} + B_{n} t)$$

where $Y_{t}$ is a univariate time sequence, and $t$ is the time point. $Z_{t}^{\prime \prime } (\theta )$ means the second-order difference of the data sequence, $\{ A_{n} ,B_{n} \}$ is the intercept and slope of $Y_{t}$.

Then,

13 $$\hat{Y}_{t} = \frac{\theta - 1}{\theta }Z_{t} (0) + \frac{1}{\theta }\hat{Z}_{t} (\theta ) = \frac{\theta - 1}{\theta }(A_{n} + B_{n} t) + \frac{1}{\theta }\hat{Z}_{t} (\theta ),\theta > 1$$

where $\hat{Y}_{t}$ is the final predicted value, and $\theta$ represents the curvature of the predicted curve.

In this paper, grey Theta residual optimization is effectively combined with fractional accumulation operator. The randomness of the original data sequence is effectively weakened by fractional accumulation, and a new GT-FGM prediction model is established.

The modeling process is described as follows:

Step 1: $r$-order accumulation generation operation.

Suppose that the original time sequence and the $r$-order accumulated data sequence are respectively $X^{(0)} = \{ x^{(0)} (1),x^{(0)} (2), \cdots ,x^{(0)} (n)\}$ and $X^{(r)} = \{ x^{(r)} (1),x^{(r)} (2), \cdots ,x^{(r)} (n)\}$, then the r-order accumulation operation is performed on the data sequence according to Eq. (1).

Step 2: $r$-order cumulative discrete equation and parameter estimation.

The discrete equation is used to describe the sequential relationship of the cumulative sequence, which is expressed as

14 $$x^{(r)} (k{ + }1) = \beta_{1} x^{(r)} (k){ + }\beta_{2} ,k = 1,2, \cdots ,n - 1$$

Then the result can be obtained by the least square method,

15 $$B = \left[ {\begin{array}{*{20}c} {x^{(r)} (1)} & 1 \\ {x^{(r)} (2)} & 1 \\ \vdots & \vdots \\ {x^{(r)} (n - 1)} & 1 \\ \end{array} } \right],\,Y = \left[ {\begin{array}{*{20}c} {x^{(r)} (2)} \\ {x^{(r)} (3)} \\ \vdots \\ {x^{(r)} (n)} \\ \end{array} } \right]$$

The system parameters can be obtained by the following formula:

16 $$[\begin{array}{*{20}c} {\beta_{1} } & {\beta_{2} } & {\beta_{3} } \\ \end{array} ]^{T} = (B^{T} B)^{ - 1} B^{T} Y$$

Step 3: Solve time response function and predicted value.

Suppose $\hat{X}^{(r)} = \{ \hat{x}^{(r)} (1),\hat{x}^{(r)} (2), \cdots ,\hat{x}^{(r)} (n), \cdots \}$ is the fitting result, $\hat{x}^{(r)} (1) = x^{(r)} (1)$, then according to formula (16), the time response function of FGM model can be obtained

17 $$\hat{x}^{(r)} (k{ + }1) = \beta_{1} \hat{x}^{(r)} (k){ + }\beta_{2}$$

Then, the predicted value $\hat{X}^{(0)} = \{ \hat{x}^{(0)} (1),\hat{x}^{(0)} (2), \cdots \}$ is the $r$-order subtractive sequence of $\hat{X}^{(r)} = \{ \hat{x}^{(r)} (1),\hat{x}^{(r)} (2), \cdots \}$. It can be defined as

18 $$\hat{x}^{(0)} (k) = \hat{x}^{(r)} (k) - \hat{x}^{(r)} (k - 1),k = 2,3, \cdots$$

Step 4: Establish Theta prediction model.

Based on the principle of "separation and combination", this paper constructs the nonlinear trend of the original data sequence by introducing $\theta$ lines, and can adjust the prediction error by curvature. The specific method is expressed as follows:

19 $$\hat{y}(k) = \frac{\theta - 1}{\theta }\hat{x}^{(0)} (k) + \frac{1}{\theta }\hat{x}_{\theta } (k)$$

where $\hat{x}^{(0)} (k)$ is the long-term trend sequence and $\hat{x}_{\theta } (k)$ represents the nonlinear trend of the local curvature of the time sequence.

$\hat{X}^{(0)}$ is supposed to be an appropriate long-term linear trend of the original sequence $X^{(0)}$. $\theta$ line is $X_{\theta } = \{ x_{\theta } (1),x_{\theta } (2), \cdots ,x_{\theta } (n)\}$, where

20 $$x_{\theta } (k) = \theta x^{(0)} (k) + (1 - \theta )\hat{x}^{(0)} (k),k = 1,2, \cdots ,n$$

Then, sequence $\hat{X}_{\theta } = \{ \hat{x}_{\theta } (1),\hat{x}_{\theta } (2), \cdots ,\hat{x}_{\theta } (n)\}$ is a simple exponential smoothing sequence of sequence $X_{\theta }$, where

21 $$\hat{x}_{\theta } (k + 1) = \alpha x_{\theta } (k) + (1 - \alpha )\hat{x}_{\theta } (k),k = 1,2, \cdots ,n$$

Simple exponential smoothing sequence selects parameter $\alpha$ by minimizing the average absolute error.

Step 5: Evaluate the prediction error of the model.

Error analysis is an important criterion to judge the accuracy of the model. The model needs to be verified to judge the reliability of prediction before application. In practical application, various methods can be used to analyze the error of the model. The parameter $\theta$ is used to adjust the curvature parameters of curves. Unreasonable parameters will produce bad prediction results. In order to evaluate the prediction accuracy of the model, this paper uses three error indicators: mean absolute error (MAE), mean absolute percentage error (MAPE) and root mean square error (RMSE) to evaluate the effectiveness of the model.

22 $$MAE = \frac{1}{n}\sum\limits_{i = 1}^{n} {\left| {\hat{y}(i) - x^{(0)} (i)} \right|}$$

23 $$MAPE = \frac{1}{n}\sum\limits_{i = 1}^{n} {\frac{{\left| {\hat{y}(i) - x^{(0)} (i)} \right|}}{{x^{(0)} (i)}}} \times 100\%$$

24 $$RMSE = \sqrt {\frac{1}{n}\sum\limits_{i = 1}^{n} {(\hat{y}(i) - x^{(0)} (i))^{2} } }$$

where $x^{(0)} (i)$ is the observed value, $\hat{y}(i)$ is the predicted result, and $n$ is the sample size.

### Hyperparameter optimization of GT-FGM by particle swarm optimization

The GT-FGM model has two hyperparameters $r,\theta$, and a particle swarm algorithm is used to optimize the hyperparameters. In order to simplify the calculation, the average absolute error within the sample is used to select the appropriate $\theta$. Effective parameter selection can save calculation time and reduce prediction error. Therefore, a simple optimization problem is established to express the principle of selecting parameters as follows:

25 $$\mathop {\min }\limits_{r,\theta } MAE = \frac{1}{n}\sum\limits_{i = 1}^{n} {\left| {\hat{y}(i) - x^{(0)} (i)} \right|}$$

26 $$s.t.\left\{ \begin{gathered} \hat{x}^{(r)} (k{ + }1) = \beta_{1} \hat{x}^{(r)} (k){ + }\beta_{2} \hfill \\ \hat{x}^{(0)} (k) = \hat{x}^{(r)} (k) - \hat{x}^{(r)} (k - 1),k = 2,3, \cdots \hfill \\ \hat{y}(k) = \frac{\theta - 1}{\theta }\hat{x}^{(0)} (k) + \frac{1}{\theta }\hat{x}_{\theta } (k) \hfill \\ x_{\theta } (k) = \theta x^{(0)} (k) + (1 - \theta )\hat{x}^{(0)} (k) \hfill \\ 1 < \theta \le 5 \hfill \\ \end{gathered} \right.$$

For this kind of nonlinear optimization problem, heuristic algorithm is often more effective than function method. Particle Swarm Optimization (PSO) is a common and easy-to-understand algorithm with low computational complexity.

For the grey prediction model with fractional order accumulation, the order will affect the prediction accuracy of the model to some extent. In order to make the prediction error of the model as small as possible, it is necessary to select the optimal order $r$. Then the optimal order can be determined by particle swarm optimization. Its basic process is to suppose that there are m particles in D-dimensional space, and the position of each particle represents a possible solution. The position parameter of the i particle is $X_{i} = (x_{i}^{1} ,x_{i}^{2} , \cdots ,x_{i}^{D} )$, and the speed parameter is $V_{i} = (v_{i}^{1} ,v_{i}^{2} , \cdots ,v_{i}^{D} )$, the best position through which it passes is the extreme value of the individual $p_{best}$. In the whole particle swarm search process, the best searched position is $g_{best}$. In each iteration, the particle velocity is updated by a single extreme value and a global extreme value. The calculation formula of particle velocity change is

27 $$V_{i + 1} = wv_{i}^{d} = c_{1} r_{1} (p_{i}^{d} - x_{i}^{d} ) + c_{2} r_{2} (p_{g}^{d} - x_{i}^{d} )$$

where $V_{i + 1}$ represents the updated particle velocity, and $w$ represents the inertia vector. $r_{1}$ and $r_{2}$ are random numbers varying in the range of [0, 1]. $c_{1}$ and $c_{2}$ are acceleration constants (usually $c_{1} = c_{2} = 2$), and $v_{i}$ is limited by the maximum speed $v_{\max }$.

In each iteration, the position of each particle is updated by the position vector and the velocity vector. The formula for determining the position of the particle is

28 $$x_{i + 1} = x_{i} + v_{i}$$

where $x_{i + 1}$ is the position of the updated particle.

**Fig. 1:**
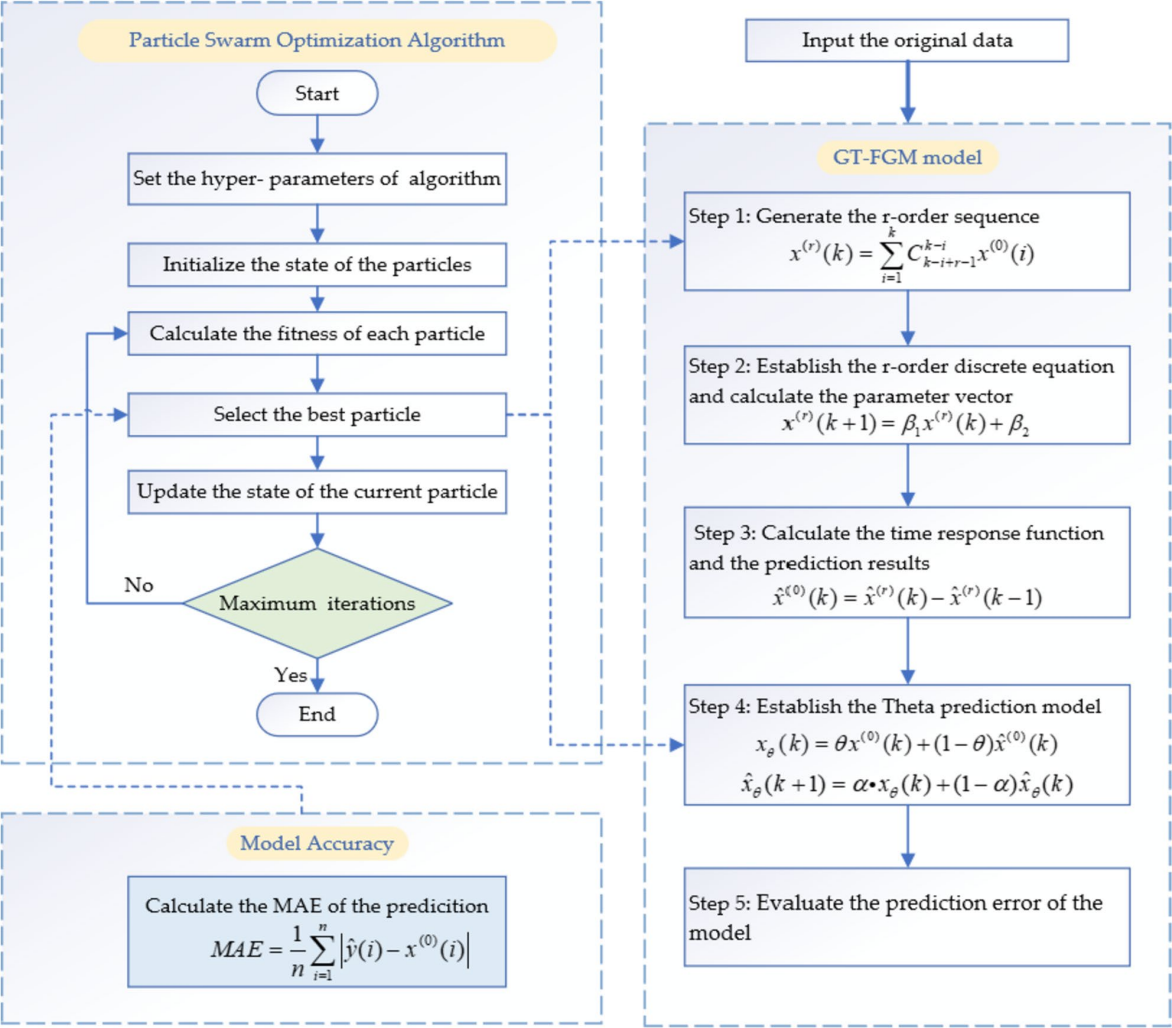
Modeling flow chart of GT-FGM model

The modeling process of GT-FGM model is shown in Fig. 1.

### Property analysis of GT-FGM

The traditional grey prediction model is sensitive to the initial values, and changes in the initial values in the original data series will not affect the fitted values of the model, and the growth rate of the prediction results will not change. This indicates that the traditional grey model has certain limitations and does not make full use of the new information and the value of information in the residuals. Based on the residual optimization method, we propose the GT-FGM model and use the heuristic algorithm to optimize the hyperparameters of the model, and the proposed model has excellent adaptability.

The innovative nature of the proposed GT-FGM model can be demonstrated by the following properties:

1. The new model has new information priority principle and the new data has a greater effect on the prediction results.

29 $$x^{(r)} (k) = \sum\limits_{i = 1}^{k} {\frac{\Gamma (r + k - i)}{{\Gamma (k - i + 1)\Gamma (r)}}x^{(0)} (i)}$$

According to Eq. (29) it can be seen that, when $r \in (0,1)$, the r-order cumulative generating operator satisfies the new information priority principle. In the expression for $x^{(r)} (k)$, the coefficient of $x^{(0)} (i)$ is larger than $x^{(0)} (i - 1)$ and has greater weight, thus satisfying the new principle of information priority.

(2) The parameter $r$ is used to adjust the data accumulation weights of the model.

When $r > 1$, the r-order cumulative generating operator satisfies the old information priority principle. In the expression for $x^{(r)} (k)$, the coefficient of $x^{(0)} (i - 1)$ is larger than $x^{(0)} (i)$ and has greater weight, thus satisfying the old principle of information priority. When $r = 1$, $a_{i - 1} = a_{i}$. In the expression for $x^{(r)} (k)$, $x^{(0)} (i - 1)$ has the same weight coefficient as $x^{(0)} (i)$.

(3) The model can use $\theta$ adaptively to adjust the effect of the fitted and true results on the predicted results. According to Eq. (20), it can be seen that the closer $\theta$ is to 1, the closer the predicted result is to the true value.
(4) The residuals of the grey model are optimized. If the trend of the original data series is not obvious, the simulation performance of the grey model is weak. And in the face of large residuals, Eq. (25) can adaptively give a smaller value of $\theta$ to avoid higher-than-expected prediction results, which improves the robustness of the model.

## Results

### Reasons for the selection of Shanghai sample data

According to the data from the National Bureau of Statistics, by the end of 2019, the elderly population aged 65 and above in China was 176.030 million, and the old-age dependency ratio was 17.8%. And by the end of 2020, the elderly population aged 60 and above in China had reached 264.018 million, among which the elderly population aged 65 and above reached 196.635 million. China's population aging is becoming increasingly severe. It is estimated that by 2025, the number of elderly population over 60 years old will reach 300 million, and China will also become a super-aged country [28]. According to the predict of the United Nations, by 2050, China's elderly population over 60 years old will account for 35% of the total population, making it the country with the most elderly population. With the improvement of economic level and the prolongation of life expectancy, the elderly population also shows a trend of aging.

However, China's population distribution and the degree of aging is uneven between regions. Therefore, it is particularly important to choose typical cases to analyze the problem of population aging. This paper takes the population data of Shanghai as a sample for case analysis, mainly for the following reasons:

1. As a first-tier city in China, the aging problem of Shanghai is more prominent than that of other cities, and the aging population data is more typical. As a city with high degree of economic development and population density, its population aging has certain particularity. According to data released by the Shanghai municipal government, the city's elderly population aged 65 and above will account for about 22.5 percent of the total population by 2025, and the elderly population is growing at a fast pace, making Shanghai an important city to study the aging problem.
2. The aging of population in Shanghai is higher than the national average level. Shanghai is the earliest city in China to enter the aging society, and also the largest large city with the highest degree of population aging. Shanghai is the economic and financial center of our country. The economy grows faster, and the proportion of elderly population in total population is higher than the national average. According to the National Bureau of Statistics, in 2022, the number of senior citizens aged 60 and above and 65 and above in Shanghai will account for 19.8 percent and 14.9 percent of China's total population, respectively.
3. The data of Shanghai are more comprehensive and reliable. As Shanghai is an international metropolis, data collection, collation and disclosure are relatively standardized and scientific. This also makes the study of the aging problem of Shanghai has a certain representative and reference value. The relatively comprehensive population data provides abundant information and data basis for the prediction of the elderly population. Shanghai has a perfect data collection system and data release mechanism, and the data quality is high, which provides strong data support for the aging population prediction and analysis. Therefore, Shanghai as a national representative of the aging population problem research, can provide reference and inspiration for other cities.
4. Compared with other areas in China, the distribution of the elderly population in Shanghai is relatively balanced, which can represent the general situation of the elderly population in China. The population distribution is more concentrated, and the sample data is more representative, which can accurately reflect the changing trend of the elderly population. At the same time, Shanghai has accumulated certain research experience and achievements in the aging problem research. Shanghai Municipal government and research institutions have carried out a series of researches and practices on the aging problem, and the achievements can provide important reference and reference.

Therefore, as one of the regions with the deepest degree of population aging, the characteristics of the aging population in Shanghai are representative and forward-looking. It is representative and feasible to choose the population sample data of Shanghai to predict the aging population. The study of Shanghai as a starting point not only has guiding significance for the change trend of Shanghai's industrial structure and relevant policy formulation, but also can serve as a reference for the future population policy formulation. It also helps to actively deal with the problem of population aging and promote the stable development of the aging industry.

### Data sources

According to data from the Shanghai Bureau of Statistics, by 2020, there will be 14.756 million people in Shanghai. Among them, there are 1.511 million elderly people aged 60–64 and 3.825 million elderly people aged 65 or above. The aging rate of the population has reached 25.9% (the proportion of people aged 65 or above in the total population). The number of the elderly population in Shanghai is gradually increasing, from 4.360 million in 2015 to 5.335 million in 2020, with an average annual growth of 302,100. The proportion of the elderly in the total population also increased from 30.21% to 36.15%, an annual increase of 1.19 percentage points. It can be seen that the degree of population aging is deepening. The degree of population aging in Shanghai is at a relatively high level in China, which is representative to a certain extent. It is not only the region with the slowest population growth rate in China, but also the region with the most serious population aging. The aging rate of Shanghai is higher than that of other major cities in China, and it is also at a high level compared with international big cities. The aging of population in Shanghai presents the following characteristics: high degree of aging, serious aging phenomenon, aging coefficient increasing year by year, aging rate of registered permanent population is significantly higher than that of the whole city, elderly migrant population shows an increasing trend, and there is a large gap between aging and social development level.

This paper takes Shanghai population data as an example to predict and analyze the total population and the number of elderly population in each district of Shanghai. The total population of each district in Shanghai from 2006 to 2020 is used, and the data source is the "Shanghai Statistical Yearbook 2021" [29]. The population data of the 16 districts in Shanghai is shown in Table 1.

**Table 1:** The permanent resident population in Shanghai's districts from 2006 to 2020 (Unit: 10^4^ people)

Year	Huangpu	Xuhui	Changning	Jing'an	Putuo	Hongkou	Yangpu	Minhang
2006	91.56	88.75	61.42	100.82	85.97	78.70	107.75	85.83
2007	91.82	89.22	61.23	100.63	86.33	78.83	107.18	88.56
2008	91.41	89.93	61.31	100.31	86.80	79.06	107.91	91.42
2009	90.99	90.64	61.39	99.98	87.27	79.28	108.63	94.28
2010	90.63	91.09	61.61	99.72	87.89	79.06	109.16	96.75
2011	90.56	91.46	62.05	99.12	88.11	79.05	109.23	98.48
2012	90.36	91.69	62.65	98.79	88.38	79.00	109.32	100.12
2013	89.77	91.70	62.50	98.04	88.58	78.74	108.95	101.97
2014	88.71	91.82	59.24	97.35	89.26	78.30	108.86	104.52
2015	87.39	91.97	58.59	96.53	89.47	77.25	108.48	106.58
2016	86.12	92.08	58.33	95.00	89.61	75.97	107.85	109.08
2017	84.59	92.12	58.12	93.71	89.53	74.46	107.63	111.40
2018	83.05	92.16	57.91	92.41	89.44	72.95	107.41	113.71
2019	80.89	92.67	57.59	91.41	89.40	71.09	106.70	116.45
2020	78.11	93.20	57.41	90.53	89.29	69.43	105.65	119.19
**Year**	**Baoshan**	**Jiading**	**Pudongxin**	**Jinshan**	**Songjiang**	**Qingpu**	**Fengxian**	**Chongming**
2006	81.59	53.25	260.29	52.29	53.21	45.63	51.33	69.98
2007	83.10	53.79	264.61	52.20	54.20	45.70	51.64	69.82
2008	84.77	54.41	268.45	51.97	55.07	45.82	51.76	69.42
2009	86.43	55.02	272.28	51.73	55.94	45.94	51.88	69.02
2010	88.29	55.75	275.80	51.66	57.60	46.19	52.18	68.95
2011	89.51	56.21	278.53	51.68	57.92	46.33	52.35	68.77
2012	90.65	56.71	281.12	51.70	58.88	46.50	52.53	68.54
2013	91.97	57.61	283.79	51.70	59.54	46.74	52.53	68.21
2014	93.60	58.83	288.44	51.79	60.57	46.94	52.69	67.78
2015	94.80	59.81	291.87	51.83	61.15	47.17	52.85	67.23
2016	96.46	61.21	295.77	52.04	62.43	47.78	53.19	67.07
2017	97.90	62.64	299.66	52.20	63.51	48.35	53.53	67.47
2018	99.33	64.07	303.54	52.36	64.59	48.92	53.87	67.86
2019	100.98	65.78	308.10	52.50	65.95	49.62	54.19	67.85
2020	102.63	67.49	312.66	52.64	67.31	50.32	54.51	67.84

**Fig. 2:**
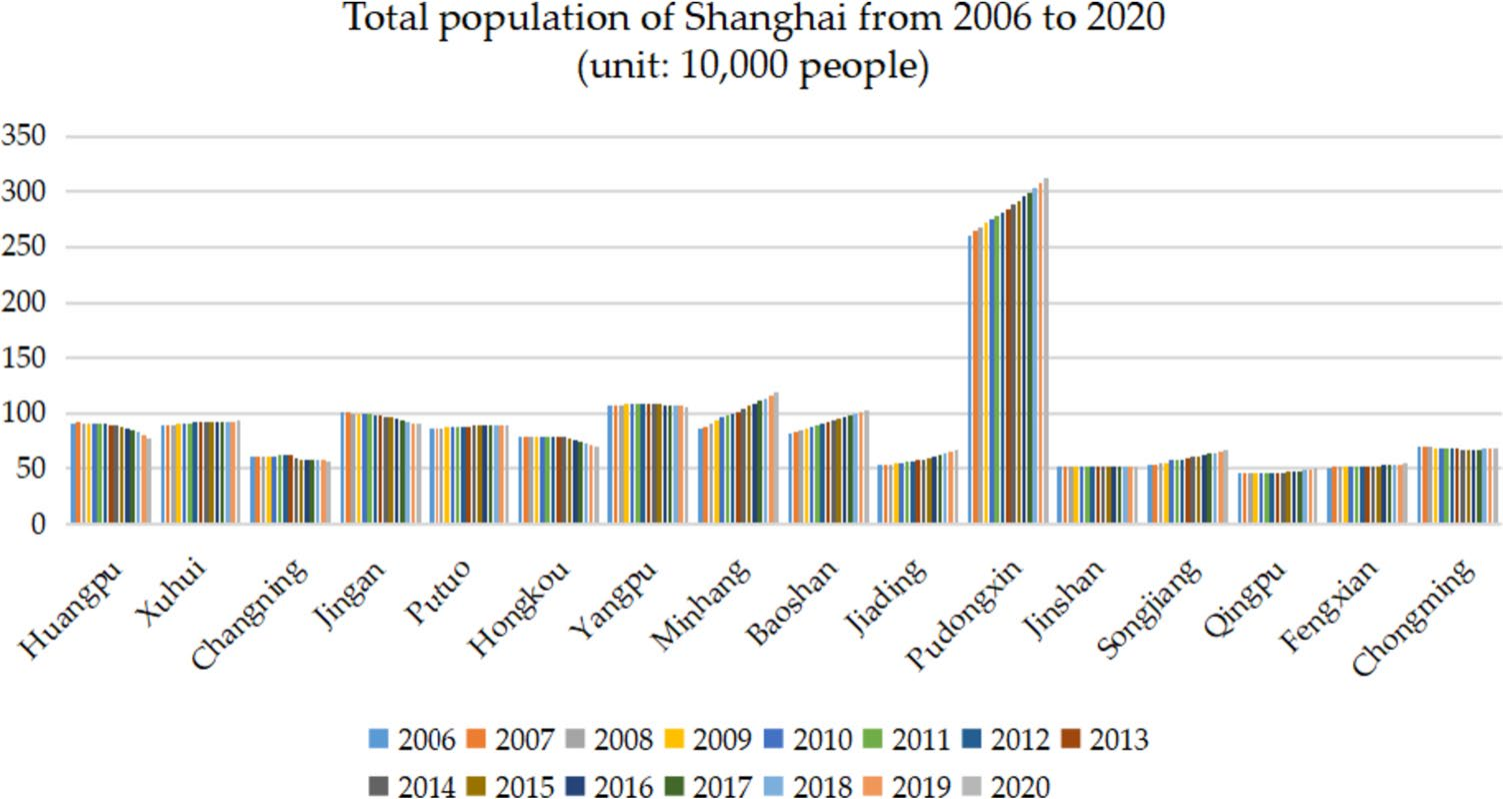
The total population of each district in Shanghai from 2006 to 2020

From the bar chart, we can intuitively see the population of each district in Shanghai from 2006 to 2020 (as shown in Fig. 2), and the total population generally shows a slight upward trend. Among them, the population of Huangpu, Changning, Jing'an, and Hongkou District showed a downward trend. The population of Xuhui, Putuo, Yangpu, Jinshan, Qingpu, Fengxian, and Chongming District changed steadily, and the fluctuation is not large, while the five districts of Minhang, Baoshan, Jiading, Pudongxin and Songjiang District show an upward trend year by year.

The statistics of the elderly population over 60 years old in each district of Shanghai are shown in Table 2. The number of the elderly population in each district is increasing, and the trend of population aging is also more obvious.

**Table 2:** The number of elderly population in each district of Shanghai from 2006 to 2020 (unit: 10^4^ people)

Year	Huangpu	Xuhui	Changning	Jing'an	Putuo	Hongkou	Yangpu	Minhang
2006	19.78	19.19	12.54	21.43	17.58	16.86	21.25	16.57
2007	20.30	19.89	12.88	22.00	18.30	17.47	21.97	17.57
2008	21.07	20.77	13.39	22.82	19.31	18.31	23.12	18.86
2009	21.84	21.65	13.89	23.63	20.32	19.15	24.27	20.14
2010	22.60	22.47	14.46	24.70	21.47	19.95	25.46	21.48
2011	23.65	23.40	15.08	25.78	22.68	20.97	26.77	22.78
2012	24.82	24.40	15.78	27.16	24.12	22.10	28.32	24.22
2013	26.05	25.47	16.52	28.40	25.66	23.31	29.92	25.78
2014	27.69	26.95	17.57	30.23	27.68	24.82	32.08	27.77
2015	28.89	28.18	18.48	31.57	29.38	25.94	33.75	29.55
2016	30.16	29.42	19.42	32.65	31.12	27.00	35.48	31.30
2017	31.38	30.72	20.40	33.87	32.86	28.03	37.36	33.09
2018	32.60	32.01	21.37	35.09	34.59	29.06	39.23	34.87
2019	32.77	32.78	21.88	35.71	35.65	29.24	40.21	36.35
2020	32.56	33.48	22.42	36.29	36.68	29.48	41.00	37.83
**Year**	**Baoshan**	**Jiading**	**Pudongxin**	**Jinshan**	**Songjiang**	**Qingpu**	**Fengxian**	**Chongming**
2006	15.74	11.24	49.57	9.65	9.97	8.83	9.67	15.76
2007	16.60	11.75	52.10	10.03	10.42	9.13	10.10	16.33
2008	17.81	13.36	55.13	10.50	10.93	9.49	10.59	16.85
2009	19.01	12.96	58.15	10.97	11.43	9.84	11.08	17.37
2010	20.22	13.62	61.28	11.43	12.00	10.27	11.61	17.99
2011	21.44	14.32	64.73	11.94	12.57	10.66	12.25	18.74
2012	22.91	15.19	68.63	12.60	13.35	11.23	12.96	19.53
2013	24.51	16.10	72.72	13.25	14.11	11.84	13.69	20.28
2014	26.56	17.26	77.90	14.07	15.10	12.60	14.56	21.14
2015	28.36	18.24	82.31	14.84	16.00	13.31	15.26	21.89
2016	30.33	19.30	86.57	15.59	16.83	13.98	15.89	22.77
2017	32.45	20.39	91.03	16.26	17.72	14.69	16.62	23.73
2018	34.56	21.48	95.49	16.92	18.6	15.39	17.34	24.68
2019	36.29	22.53	98.92	17.33	19.31	15.98	17.69	25.48
2020	38.02	23.58	102.35	17.74	20.02	16.57	18.04	26.28

**Fig. 3:**
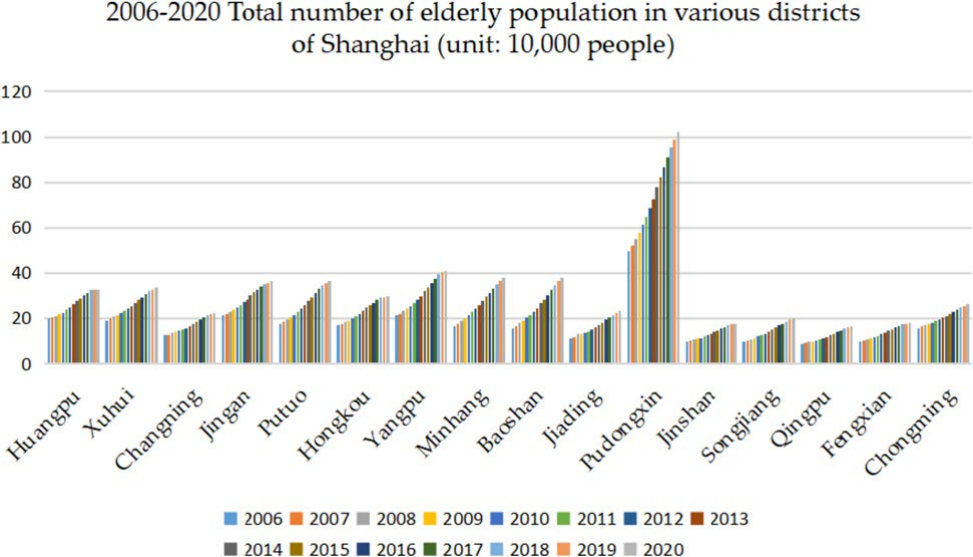
The total number of elderly population in each district of Shanghai from 2006 to 2020

From the bar chart, we can intuitively see the number of elderly population in each district in Shanghai from 2006 to 2020 (as shown in Fig. 3), and the total number of elderly population in each district shows an increasing trend year by year.

The basic age structure distribution of the elderly population in Shanghai is shown in Table 3, which includes the data of the elderly population in the three age groups of 60–69 years old, 70–79 years old and over 80 years old.

**Table 3:** Distribution of age structure of the elderly population in Shanghai from 2006 to 2020 (unit: 10^4^ people)

Year	Total population	Age			
		**60 + **	**60–69**	**70–79**	**80 + **
2006	1368.06	275.62	120.45	108.32	46.86
2007	1378.86	286.83	128.21	108.42	50.20
2008	1389.82	300.57	140.07	107.30	53.50
2009	1400.70	315.70	153.11	105.76	56.51
2010	1412.32	331.02	166.83	104.60	59.91
2011	1419.36	347.76	180.49	104.33	62.94
2012	1426.93	367.32	198.35	102.11	66.85
2013	1432.34	387.62	215.52	100.39	71.71
2014	1438.69	413.98	236.80	101.84	75.34
2015	1442.97	435.95	255.03	102.88	78.04
2016	1449.98	457.79	269.18	108.95	79.66
2017	1456.82	483.60	283.87	118.48	81.24
2018	1463.61	503.28	295.43	126.83	81.53
2019	1471.16	518.12	297.40	138.86	81.86
2020	1478.09	533.49	299.82	150.98	82.69

### Population prediction

In order to verify the validity of the model, the total population of each district in Shanghai is selected as an example. Jing'an District is one of the districts with the highest degree of aging population in Shanghai, and it is the earliest urban area in Shanghai to enter deep aging. The total population of Jing'an District shows the trend of decreasing year by year. By the end of 2020, the region has a registered population of 905,300, among which 362,900 are aged 60 or above, accounting for 40.1% of the region's total registered population, indicating a serious aging population. As the only pilot area in Shanghai for the first batch of home-based and community-based basic elderly care Service Improvement action project, Jing'an District is representative to some extent.

Firstly, taking the population prediction of Jing'an District as an example, taking the population of Jing'an District from 2006 to 2020 as a sample, and the GT-FGM model is established to predict the total population of Jing'an District from 2021 to 2030. The modeling process of Jing'an District’s population prediction is as follows:

Step 1: The original sequence of the total population of Jing'an District is

$$X^{(0)} = \{ 100.82,100.63,100.31,99.98,99.72,99.12,98.79,98.04,97.35,96.53,95.00,93.71,92.41,91.41,90.53\}$$

The PSO algorithm is used to find the optimal order $r$, which makes the error of the model minimum. The calculation results show that the order 0.9491 is the optimal order. The model error is the smallest under this order, which is 0.30%, and the prediction result is more accurate. The 0.9491th order accumulation sequence is

$$\begin{gathered} X^{(0.9491)} = \{ x^{(0.9491)} (1),x^{(0.9491)} (2),x^{(0.9491)} (3),x^{(0.9491)} (4),x^{(0.9491)} (5),x^{(0.9491)} (6),x^{(0.9491)} (7),x^{(0.9491)} (8), \hfill \\ \begin{array}{*{20}c} {} & {} & {} & {} \\ \end{array} x^{(0.9491)} (9),x^{(0.9491)} (10)x^{(0.9491)} (11),x^{(0.9491)} (12),x^{(0.9491)} (13)x^{(0.9491)} (14),x^{(0.9491)} (15)\} \hfill \\ \begin{array}{*{20}c} {} & {} & {} \\ \end{array} = \{ 100.82,196.32,289.08,379.95,469.42,557.40,644.34,729.93,814.33,897.48,978.76 \hfill \\ \begin{array}{*{20}c} {} & {} & {} & {} \\ \end{array} 1059.51,1136.70,1213.72,1289.63\} \hfill \\ \end{gathered}$$

Step 2: The values of unknown parameters $\beta_{1} ,\beta_{2}$ can be obtained by the following formula

$$\left[ \begin{gathered} \beta_{1} \hfill \\ \beta_{2} \hfill \\ \end{gathered} \right] = \left[ \begin{gathered} 0.0170 \hfill \\ 97.1287 \hfill \\ \end{gathered} \right] = (B^{T} B)^{ - 1} B^{T} Y$$

Then,

$$x^{(0.9491)} (k{ + }1) = 0.0170x^{(0.9491)} (k){ + }97.1287,k = 1,2, \cdots ,n - 1$$

Step 3: The time response function is

$$\hat{x}^{(0.9491)} (k + 1) = (97.1287 - \frac{97.1287}{{0.0170}})e^{ - 0.0170k} + \frac{97.1287}{{0.0170}}$$

The above formula can be obtained

$$\begin{gathered} \hat{X}^{(0.9491)} = \{ 100.82,195.42,288.43,379.87,469.77,558.15,645.04,730.46,814.45,897.02,978.20, \hfill \\ \begin{array}{*{20}c} {} & {} & {} \\ \end{array} \, 1058.01,1136.50,1213.62,1289.41\} \hfill \\ \end{gathered}$$

The 0.0509-order accumulation of the sequence $\hat{X}^{(0.9491)}$ can obtain the predicted value $\hat{X}^{(1)}$ of the 1-order accumulation of the original sequence as follows

$$\begin{gathered} \hat{X}^{(1)} = \{ 100.82,202.80,303.94,404.24,503.71,602.36,797.23,893.45,988.89,1083.50,1177.41 \hfill \\ \begin{array}{*{20}c} {} & {} & {} \\ \end{array} 1270.52,1362.81,1454.40\} \hfill \\ \end{gathered}$$

Then, $\hat{X}^{(1)}$ is restored, and the predicted value of the original data is

$$\hat{X}^{(0)} = \{ 100.81,101.98,101.14,100.30,99.47,98.65,97.84,97.03,96.23,95.43,94.64,93.86,93.09,92.32,92.56\}$$

Step 4: The optimal parameter $\theta$ is obtained by particle swarm optimization, and $\theta_{best} = 5$. Then

$$\hat{y}(k) = \frac{4}{5}\hat{x}^{(0)} (k) + \frac{1}{5}\hat{x}_{\theta } (k)$$

$$x_{\theta } (k) = 5x^{(0)} (k) - 4\hat{x}^{(0)} (k),k = 1,2, \cdots ,n$$

Step 5: Use the average absolute error within the sample to select the appropriate $\theta$. The optimization problem is as follows:

$$\mathop {\min }\limits_{\theta } MAE = \frac{1}{15}\sum\limits_{i = 1}^{15} {\left| {\hat{y}(i) - x^{(0)} (i)} \right|}$$

$$s.t.\left\{ \begin{gathered} x^{(0.9491)} (k{ + }1) = 0.0170x^{(0.9491)} (k){ + }97.1287,k = 1,2, \cdots ,n - 1 \hfill \\ \hat{x}^{(0)} (k) = \hat{x}^{(0.9491)} (k) - \hat{x}^{(0.9491)} (k - 1),k = 2,3, \cdots \hfill \\ \hat{y}(k) = \frac{4}{5}\hat{x}^{(0)} (k) + \frac{1}{5}\hat{x}_{\theta } (k) \hfill \\ x_{\theta } (k) = 5x^{(0)} (k) - 4\hat{x}^{(0)} (k) \hfill \\ \end{gathered} \right.$$

In order to verify the accuracy and effectiveness of the model, the population data of Jing'an District is predicted by this model. The data from 2006 to 2016 are selected as fitting samples, and the data from 2017 to 2020 are used as predicting samples to test the predicting effect of GT-FGM model. Then, MAE, MAPE and RMSE are used to test the accuracy of the model, and the predicted values of GT-FGM model, FGM model, GM(1,1) model, Verhulst model and Simple Exponential Smoothing (SES) are compared. The predicted results of Jing'an District population under different models are shown in Table 4.

**Table 4:** Comparison of population prediction of Jing'an District under five models (unit: 10^4^ people)

**Year**	**True value**	**GT-FGM**	**FGM**	GM(1,1)	Verhulst	SES
2006	100.82	100.82	100.82	100.82	100.75	100.82
2007	100.63	100.65	100.61	101.17	100.58	100.82
2008	100.31	100.57	100.54	100.57	100.36	100.63
2009	99.98	100.02	100.18	99.99	100.09	100.31
2010	99.72	99.58	99.68	99.40	99.75	99.98
2011	99.12	99.25	99.10	98.82	99.32	99.72
2012	98.79	98.61	98.46	98.24	98.78	99.12
2013	98.04	98.25	97.79	97.67	98.11	98.79
2014	97.35	97.48	97.09	97.10	97.26	98.04
2015	96.53	96.78	96.37	96.53	96.21	97.35
2016	95.00	95.95	95.64	95.97	94.92	96.53
**MAE**		0.21	0.20	0.32	**0.10**	0.53
**MAPE(%)**		0.22%	0.20%	0.33%	**0.10%**	0.54%
**RMSE**		0.32	0.26	0.43	**0.13**	0.66
2017	93.71	94.41	94.90	95.41	93.32	95.00
2018	92.41	93.81	94.16	94.85	91.38	95.00
2019	91.41	93.21	93.41	94.30	89.02	95.00
2020	90.53	92.61	92.66	93.74	86.21	95.00
**MAE**		**1.49**	1.77	2.56	2.03	2.99
**MAPE(%)**		**1.63%**	1.93%	2.79%	2.23%	3.26%
**RMSE**		**1.58**	1.80	2.62	2.53	3.21

Data in boldface highlights the prediction errors of the corresponding prediction model is minimal

**Fig. 4:**
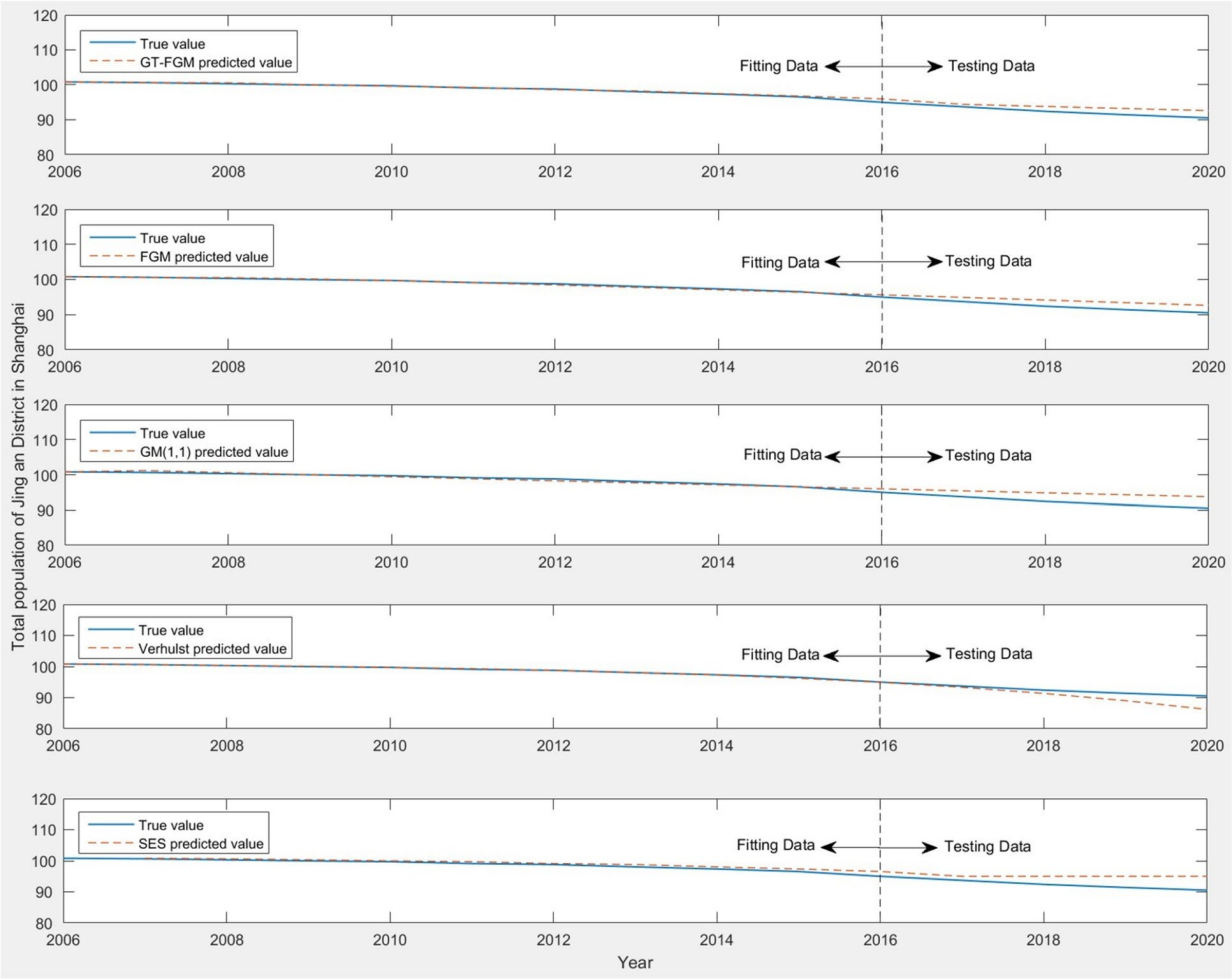
Comparison of the predicted results of the total population in Jing'an District under five models

As can be seen from Table 4, the prediction results of the five models have good performance and high prediction accuracy. The fitting error of Verhulst model is relatively small, while the prediction error of GT-FGM model is smaller than that of FGM model, GM(1,1) model, Verhulst model and SES. The average absolute percentage errors of the five models are all significantly lower than 10%, while the predicted MAPE value of GT-FGM model is 1.63%, which also indicates that this model has high multi-step prediction accuracy and can effectively predict the future trend of Shanghai population. The prediction trend curves of five different comparison models for the total population of Jing'an District is shown in Fig. 4. It is obvious that GT-FGM model has obvious overall advantages over other comparison models in both the fitting area and the testing area, especially reflecting the stable and reliable multi-step prediction ability.

The exact criteria of MAPE value are shown in Table 5, from which it can be judged whether the prediction error is accepted or not. When the MAPE value is less than 10%, the prediction performance of the model is excellent, indicating that the prediction accuracy of the model is high and the error is relatively small. The MAPE value of the predicted results in Jing'an District is only 1.63%, and the prediction error of the model is small. Therefore, the GT-FGM model has better predict performance.

**Table 5:** Accuracy criteria of MAPE values

**MAPE(%)**	< 10	10–20	20–50	> 50
**Predictive performance**	excellent	good	common	poor

From the predicted results of Jing'an District, it can be seen that the GT-FGM model proposed in this paper has a small predict error and a relatively good predict effect. It can be used to predict the population of various districts in Shanghai from 2021 to 2030. The predicted results are shown in Table 6.

**Table 6:** Predicted population of Shanghai from 2021 to 2030 (unit: 10^4^ people)

Year	Huangpu	Xuhui	Changning	Jing'an	Putuo	Hongkou	Yangpu	Minhang
2021	76.92	93.27	57.24	89.62	89.29	68.81	105.65	121.23
2022	75.73	93.33	57.08	88.70	89.28	68.19	105.31	123.30
2023	74.54	93.39	56.93	87.79	89.27	67.58	104.98	125.42
2024	73.37	93.44	56.79	86.87	89.25	66.96	104.68	127.58
2025	72.20	93.49	56.65	85.96	89.23	66.35	104.38	129.78
2026	71.04	93.53	56.53	85.05	89.20	65.74	104.10	132.03
2027	69.90	93.58	56.41	84.15	89.17	65.15	103.83	134.33
2028	68.77	93.61	56.29	83.25	89.15	64.56	103.57	136.67
2029	67.65	93.65	56.18	82.36	89.12	63.98	103.32	139.06
2030	66.55	93.68	56.08	81.47	89.08	63.40	103.09	141.50
**Year**	**Baoshan**	**Jiading**	**Pudongxin**	**Jinshan**	**Songjiang**	**Qingpu**	**Fengxian**	**Chongming**
2021	103.85	69.10	315.79	52.79	68.16	51.00	54.79	67.70
2022	105.08	70.89	318.97	52.95	69.04	51.78	55.08	67.75
2023	106.33	72.87	322.18	53.12	69.92	52.68	55.40	67.71
2024	107.59	75.07	325.44	53.31	70.82	53.71	55.73	67.68
2025	108.87	77.50	328.73	53.52	71.74	54.91	56.09	67.65
2026	110.17	80.19	332.07	53.75	72.67	56.28	56.47	67.62
2027	111.48	83.18	335.45	53.99	73.61	57.86	56.87	67.60
2028	112.81	86.49	338.87	54.26	74.57	59.69	57.31	67.57
2029	114.15	90.16	342.33	54.54	75.55	61.79	57.76	67.56
2030	115.52	94.23	345.84	54.85	76.54	64.21	58.25	67.54

As can be seen from Table 6, the population of Shanghai shows a steady growth trend. It is estimated that the total population of Shanghai will reach 15,718,300 by 2030. Among them, the population of Huangpu, Changning, Jing'an, Putuo, Hongkou, Yangpu and Chongming Districts showed a slight downward trend, while that of Xuhui, Minhang, Baoshan, Jiading, Pudongxin, Jinshan, Songjiang, Qingpu and Fengxian Districts showed an upward trend year by year. Xuhui, Changning, Putuo and Chongming Districts have not changed much in general.

**Fig. 5:**
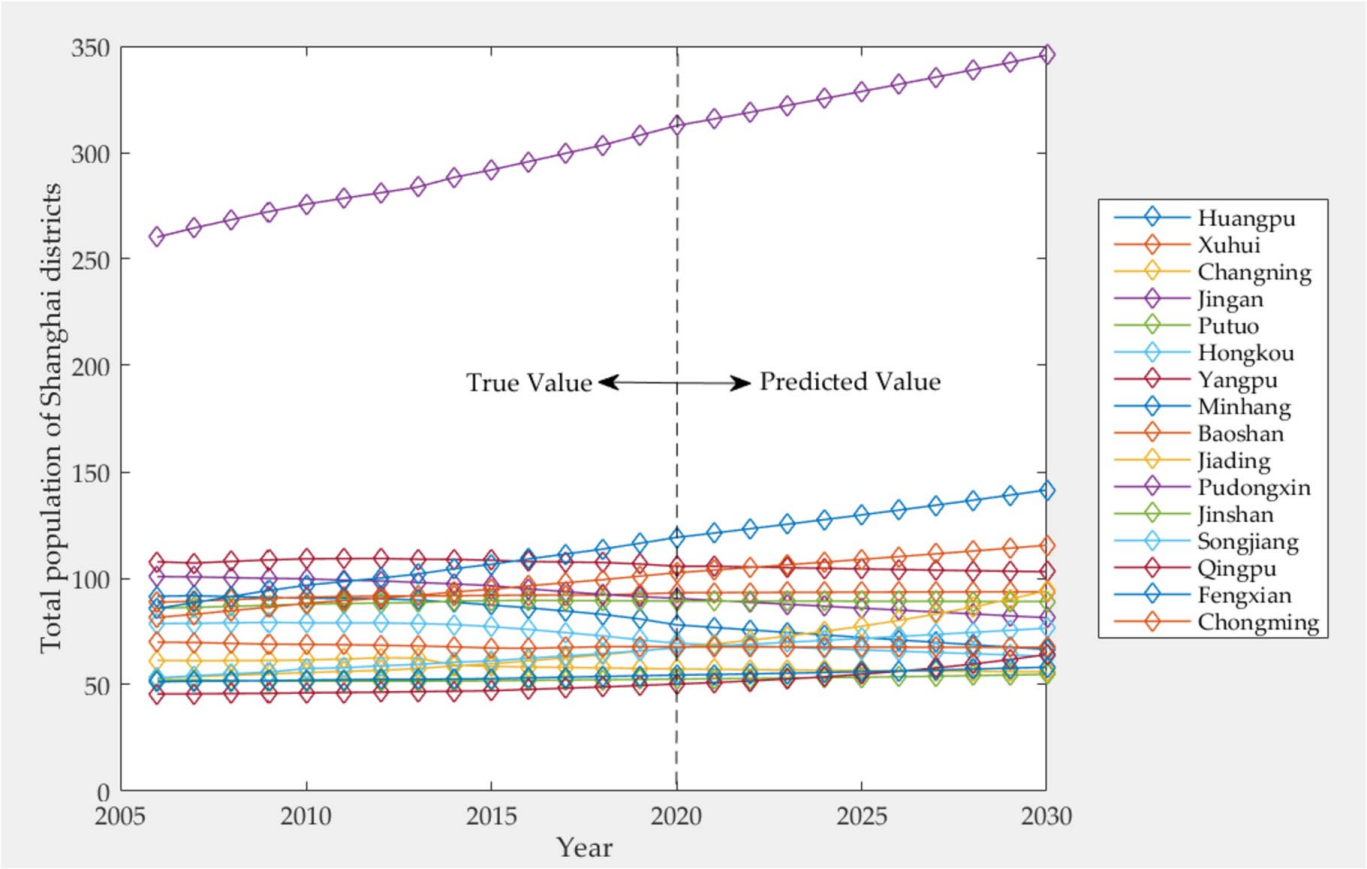
Predicted results of total population in various districts of Shanghai

According to the predicted results, by 2030, the population of each district will be 665,500 in Huangpu, 936,800 in Xuhui, 560,800 in Changning, 814,700 in Jing'an, 890,800 in Putuo, 634,000 in Hongkou, 1,030,900 in Yangpu, 1,415,000 in Minhang,1155,200 in Baoshan, 942,300 in Jiading, 3,458,400 people in Pudongxin, 548,500 in Jinshan, 765,400 in Songjiang, 642,100 in Qingpu, 582,500 in Fengxian and 675,400 in Chongming. The predicted results of the total population of each district in Shanghai are shown in Fig. 5.

## Discussion

### Predict the number and density of the elderly population

Based on the prediction of the total population of all districts in Shanghai, it is found that GT-FGM model has a relatively good prediction effect. Next, taking the total number of elderly population in Shanghai as an example, and comparing it with FGM model, GM(1,1) model, Verhulst model, Logistic model and SES, then test the prediction effect of the model. The prediction results of GM(1,1) model basically accord with the trend of exponential growth, but for complex nonlinear data sequence, its prediction effect is often not ideal. FGM model is the optimized form of GM(1,1) model, but from the test data, the prediction error is greater than GT-FGM model. Verhulst model and SES are both time series prediction models. Verhulst model is a nonlinear model based on Logistic growth model, which is suitable for time series data with S-shaped growth trend. For data series with nonlinear growth trend, the model has a better prediction effect. SES is a kind of exponential smoothing model, which is suitable for time series data with relatively stable growth trend. From the comparative analysis of the test data, the overall prediction performance of the GT-FGM model is relatively better.

**Fig. 6:**
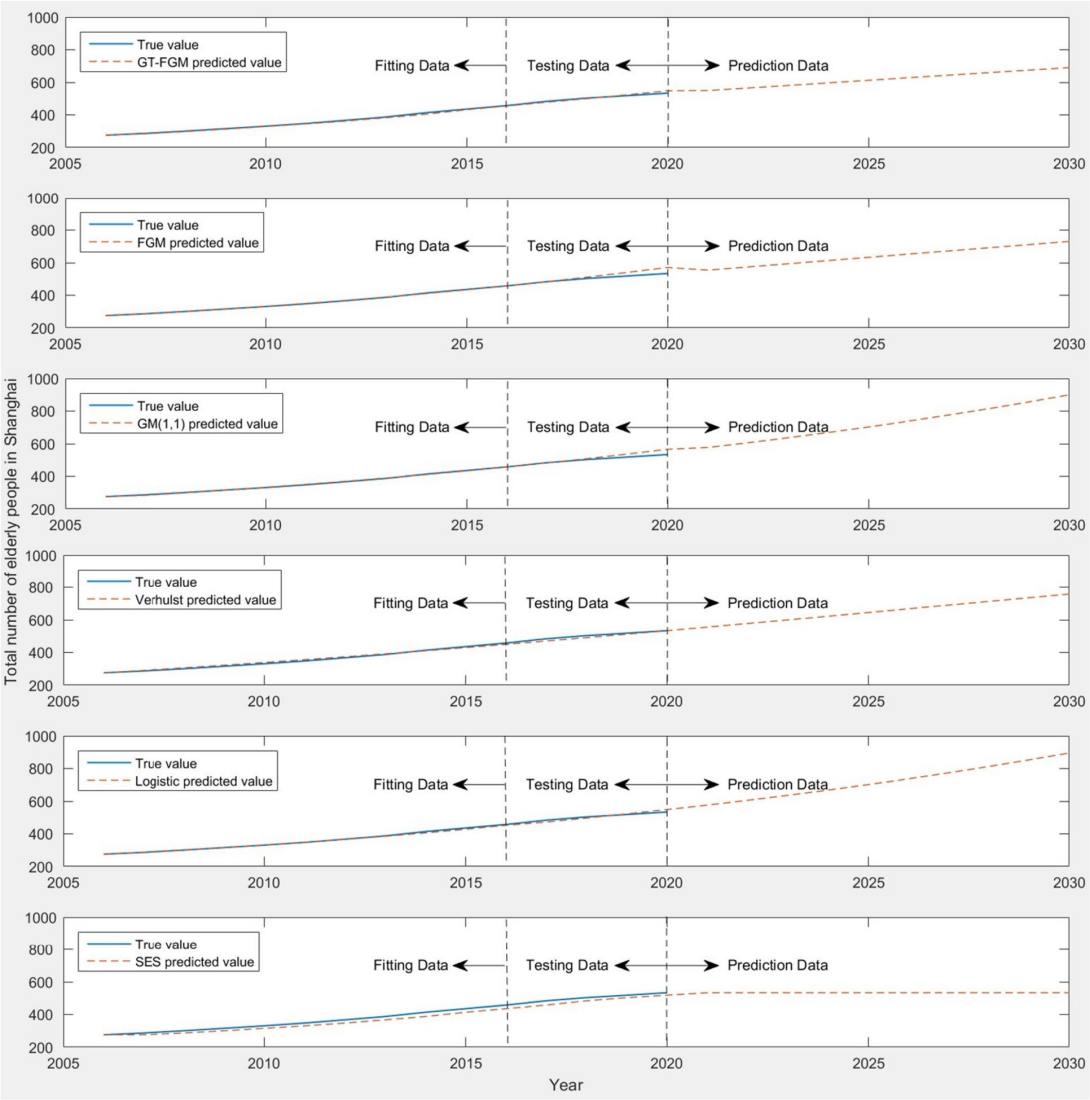
Comparison of the predicted results of the total elderly population in Shanghai under six models

Therefore, it can be seen that the test results of GT-FGM model are more in line with the real data and can effectively predict the number of elderly population in Shanghai. The comparison of the prediction results of the total elderly population in Shanghai under the six models is shown in Fig. 6.

Therefore, GT-FGM model can be used to predict the data of the elderly population in various districts of Shanghai, compared with FGM model, GM(1,1) model, Verhulst model, Logistic model and SES, and the prediction accuracy of the model can be tested with the help of three error indicators: MAE, MAPE and RMSE. Taking Jing'an District as an example, the prediction results of the elderly population are shown in Table 7.

**Table 7:** Predicted results of elderly population in Jing'an District under six models (unit: 10^4^ people)

Year	True value	GT-FGM	FGM	GM(1,1)	Verhulst	Logistic	SES
2006	21.43	21.43	21.43	21.43	21.35	21.43	21.43
2007	22.00	21.85	21.95	21.67	22.16	22.21	21.43
2008	22.82	22.69	22.82	22.69	23.02	23.04	22.00
2009	23.63	23.62	23.82	23.76	23.94	23.92	22.82
2010	24.70	24.50	24.91	24.88	24.92	24.85	23.63
2011	25.78	25.62	26.06	26.05	25.96	25.84	24.70
2012	27.16	26.75	27.27	27.27	27.08	26.90	25.78
2013	28.40	28.17	28.54	28.56	28.29	28.03	27.16
2014	30.23	29.46	29.86	29.90	29.58	29.23	28.40
2015	31.57	31.33	31.24	31.31	30.98	30.53	30.23
2016	32.65	32.71	32.67	32.78	32.50	31.92	31.57
**MAE**		0.21	**0.15**	0.18	0.25	0.39	1.02
**MAPE(%)**		0.78%	**0.56%**	0.70%	0.91%	1.37%	3.74%
**RMSE**		0.30	**0.20**	0.21	0.31	0.52	1.12
2017	33.87	33.84	34.15	34.32	34.14	34.19	32.65
2018	35.09	35.07	35.69	35.94	35.93	35.75	32.65
2019	35.71	36.35	37.29	37.63	37.89	37.39	32.65
2020	36.29	37.68	38.95	39.40	40.05	39.10	32.65
**MAE**		**0.52**	1.28	1.58	1.76	1.37	2.59
**MAPE(%)**		**1.44%**	3.57%	4.42%	4.91%	3.82%	7.29%
**RMSE**		**0.77**	1.58	1.89	2.22	1.68	2.74

Data in boldface highlights the prediction errors of the corresponding prediction model is minimal

As can be seen from Table 7, the fitting and prediction error indexes of the six comparison models are all small. The fitting error of the FGM model is the smallest, while the prediction error of the GT-FGM model is the smallest, and its MAPE value reaches 1.44%, indicating that the multi-step prediction effect of the GT-FGM model is relatively optimal. The results show that the GT-FGM model has high prediction accuracy and can effectively predict the number of elderly population in Shanghai.

Next, the elderly population prediction of Xuhui District is also taken as an example for verification. The prediction results of the six comparison models are shown in Table 8.

**Table 8:** Predicted results of elderly population in Xuhui District under six models (unit: 10^4^ people)

Year	True value	GT-FGM	FGM	GM(1,1)	Verhulst	Logistic	SES
2006	19.19	19.19	19.19	19.19	19.18	19.19	19.19
2007	19.89	19.79	19.94	19.76	19.94	19.93	19.19
2008	20.77	20.53	20.74	20.64	20.74	20.71	19.89
2009	21.65	21.45	21.59	21.57	21.59	21.55	20.77
2010	22.47	22.38	22.50	22.53	22.50	22.43	21.65
2011	23.40	23.25	23.48	23.54	23.47	23.37	22.47
2012	24.40	24.23	24.51	24.60	24.50	24.38	23.40
2013	25.47	25.29	25.62	25.70	25.60	25.45	24.40
2014	26.95	26.42	26.81	26.85	26.77	26.60	25.47
2015	28.18	27.96	28.07	28.05	28.04	27.84	26.95
2016	29.42	29.26	29.42	29.31	29.40	29.17	28.18
**MAE**		0.18	**0.07**	0.12	**0.07**	0.11	0.93
**MAPE(%)**		0.76%	**0.28%**	0.49%	0.30%	0.43%	3.80%
**RMSE**		0.22	**0.09**	0.13	**0.09**	0.17	1.00
2017	30.72	30.57	30.86	30.62	30.87	30.57	29.42
2018	32.01	31.80	32.40	31.99	32.45	31.92	29.42
2019	32.78	33.12	34.05	33.42	34.18	33.34	29.42
2020	33.48	34.52	35.80	34.92	36.05	34.81	29.42
**MAE**		**0.44**	1.03	0.55	1.14	0.53	2.83
**MAPE(%)**		**1.32%**	3.12%	1.66%	3.45%	1.61%	8.67%
**RMSE**		**0.56**	1.34	0.79	1.48	0.73	3.01

Data in boldface highlights the prediction errors of the corresponding prediction model is minimal

Similar to the fitting and prediction results of Jing'an District, the fitting errors of FGM model and Verhulst model are relatively small, while the multi-step prediction errors of GT-FGM model are relatively minimal. The MAPE value of GT-FGM model is as low as 1.32%, indicating that the prediction effect of this model is relatively optimal.

Next, taking the number of elderly population in each district of Shanghai from 2006 to 2020 as the original data sequence, and adopting the same modeling steps as above, the predicted number of elderly population in each district of Shanghai from 2021 to 2030 is obtained, as shown in Table 9.

**Table 9:** Predicted number of elderly population in Shanghai from 2021 to 2030 (unit: 10^4^ people)

Year	Huangpu	Xuhui	Changning	Jing'an	Putuo	Hongkou	Yangpu	Minhang
2021	33.64	34.63	23.27	37.02	37.89	30.03	42.69	39.22
2022	33.76	35.83	24.16	37.72	39.11	30.56	44.46	40.62
2023	35.93	37.07	25.09	38.39	40.33	31.06	46.32	42.03
2024	37.14	38.37	26.07	39.03	41.55	31.53	48.27	43.47
2025	38.39	39.73	27.09	39.65	42.77	31.98	50.31	44.91
2026	39.69	41.13	28.16	40.24	44.00	32.40	52.45	46.38
2027	41.04	42.60	29.27	40.81	45.23	32.81	54.70	47.86
2028	42.45	44.13	30.44	41.36	46.46	33.19	57.06	49.35
2029	43.90	45.72	31.66	41.88	47.70	33.55	59.53	50.86
2030	45.42	47.38	32.94	42.38	48.94	33.89	62.12	52.39
**Year**	**Baoshan**	**Jiading**	**Pudongxin**	**Jinshan**	**Songjiang**	**Qingpu**	**Fengxian**	**Chongming**
2021	40.06	24.51	105.66	18.41	20.89	17.13	18.46	27.10
2022	42.24	25.48	108.98	19.11	21.82	17.71	18.87	27.94
2023	44.55	26.47	112.31	19.85	22.79	18.30	19.26	28.82
2024	47.02	27.50	115.65	20.62	23.81	18.90	19.64	29.73
2025	49.65	28.56	119.00	21.42	24.89	19.51	20.01	30.68
2026	52.45	29.65	122.35	22.26	26.02	20.13	20.37	31.67
2027	55.43	30.78	125.72	23.13	27.22	20.77	20.72	32.69
2028	58.61	31.94	129.10	24.05	28.48	21.42	21.05	33.75
2029	61.99	33.15	132.49	25.01	29.81	22.08	21.37	34.85
2030	65.60	34.39	135.89	26.01	31.21	22.75	21.69	36.00

**Fig. 7:**
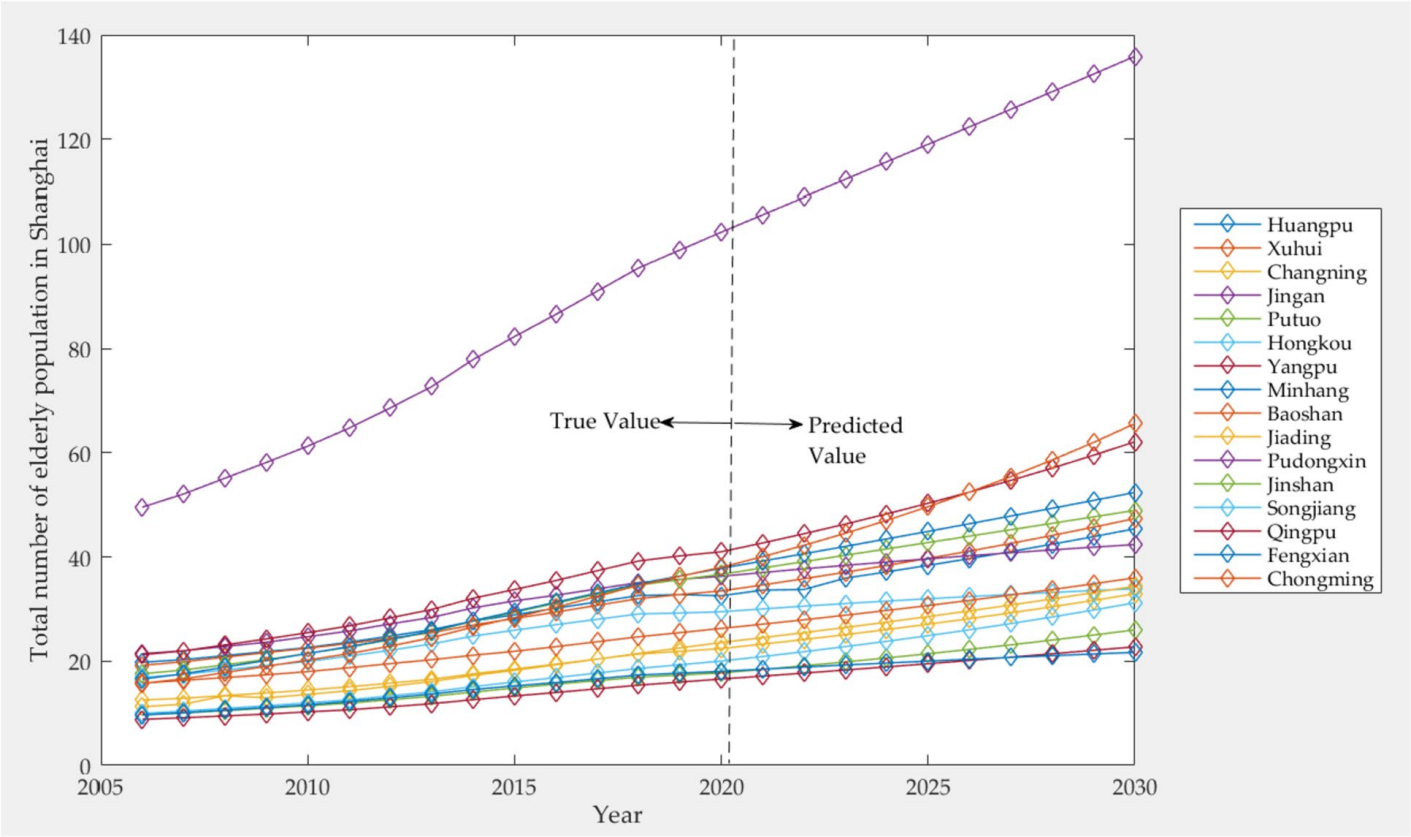
Predicted results of total elderly population in various districts of Shanghai

As can be seen from Table 9, the number of elderly population in all districts of Shanghai is increasing continuously. It is estimated that the total number of elderly population in Shanghai will reach 7,391,000 by 2030, while the number of elderly population in Pudongxin District is the largest, reaching 1,358,900. The predicted total number of elderly population in all districts of Shanghai shows a gradual growth trend, as shown in Fig. 7.

According to the above-mentioned total population and the number of elderly population in various districts of Shanghai from 2021 to 2030, the density of elderly population in various districts of Shanghai can be obtained, as shown in Table 10.

**Table 10:** Predicted values of elderly population density in Shanghai from 2021 to 2030 (unit: %)

Year	Huangpu	Xuhui	Changning	Jing'an	Putuo	Hongkou	Yangpu	Minhang
2021	43.73	37.13	40.65	41.31	42.43	43.64	40.41	32.35
2022	44.58	38.39	42.33	42.53	43.81	44.82	42.22	32.94
2023	48.20	39.69	44.07	43.73	45.18	45.96	44.12	33.51
2024	50.62	41.06	45.91	44.93	46.55	47.09	46.11	34.07
2025	53.17	42.50	47.82	46.13	47.93	48.20	48.20	34.60
2026	55.87	43.98	49.81	47.31	49.33	49.29	50.38	35.13
2027	58.71	45.52	51.89	48.50	50.72	50.36	52.68	35.63
2028	61.73	47.14	54.08	49.68	52.11	51.41	55.09	36.11
2029	64.89	48.82	56.35	50.85	53.52	52.44	57.62	36.57
2030	68.25	50.58	58.74	52.02	54.94	53.45	60.26	37.02
**Year**	**Baoshan**	**Jiading**	**Pudongxin**	**Jinshan**	**Songjiang**	**Qingpu**	**Fengxian**	**Chongming**
2021	38.57	35.47	33.46	34.87	30.65	33.59	33.69	40.03
2022	40.20	35.94	34.17	36.09	31.60	34.20	34.26	41.24
2023	41.90	36.32	34.86	37.37	32.59	34.74	34.77	42.56
2024	43.70	36.63	35.54	38.68	33.62	35.19	35.24	43.93
2025	45.60	36.85	36.20	40.02	34.69	35.53	35.67	45.35
2026	47.61	36.97	36.84	41.41	35.81	35.77	36.07	46.84
2027	49.72	37.00	37.48	42.84	36.98	35.90	36.43	48.36
2028	51.95	36.93	38.10	44.32	38.19	35.89	36.73	49.95
2029	54.31	36.77	38.70	45.86	39.46	35.73	37.00	51.58
2030	56.79	36.50	39.29	47.42	40.78	35.43	37.24	53.30

**Fig. 8:**
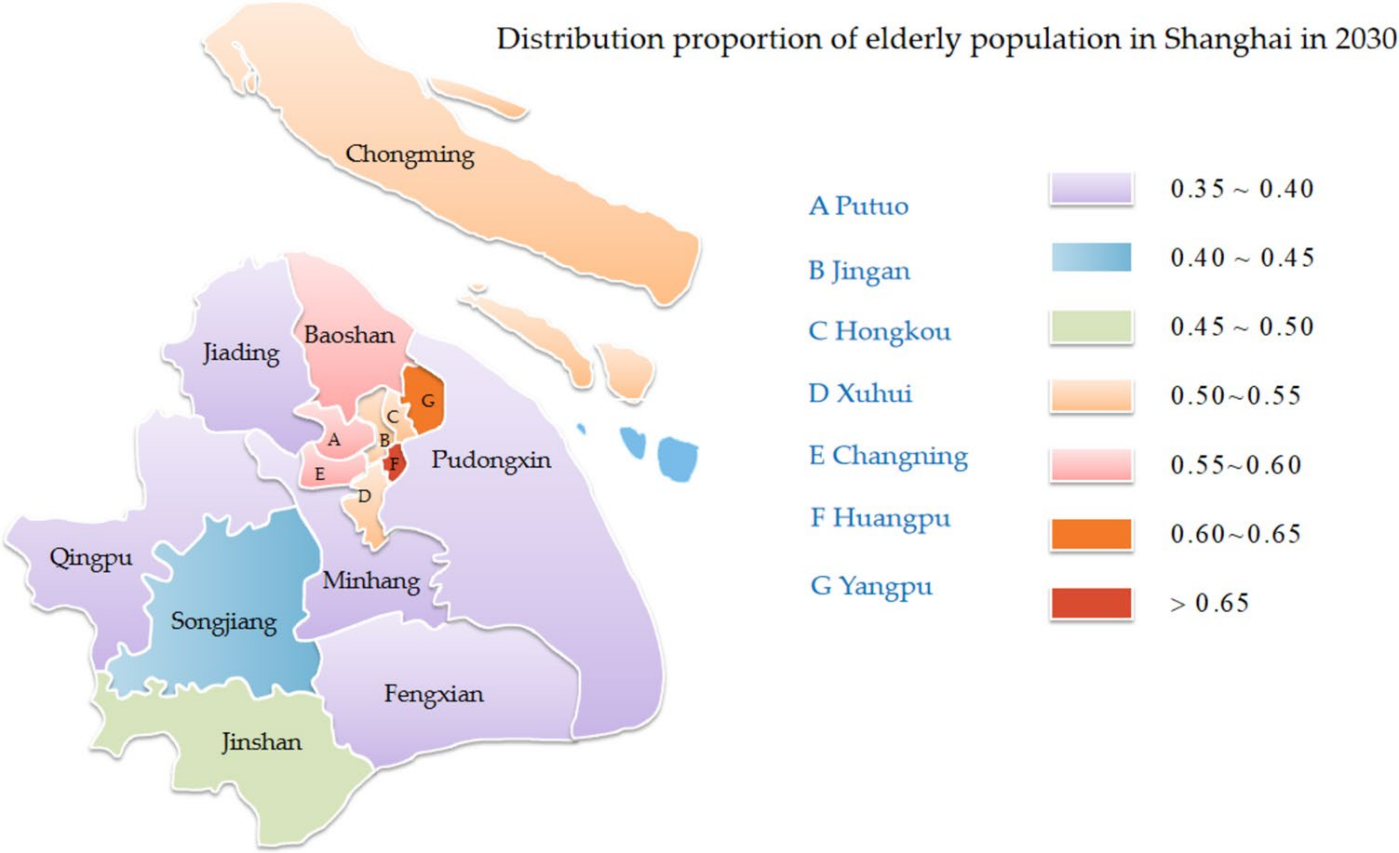
Density distribution of elderly population in Shanghai in 2030

It can be seen from Table 10 that the elderly population density in Huangpu District of Shanghai will be the highest by 2030, reaching 0.6825, while that in Qingpu District will be the lowest, reaching 0.3543. It is predicted that the elderly population density in nine districts of Shanghai will exceed 0.5, including Huangpu, Xuhui, Changning, Jing'an, Putuo, Hongkou, Yangpu, Baoshan and Chongming Districts. Through the map marking method [30], the distribution of the elderly population density in Shanghai in 2030 was drawn, as shown in Fig. 8.

### Predicting the age structure of the elderly population

Next, the GT-FGM model is used to predict the basic situation of the age structure of the elderly population in Shanghai. The prediction results of the elderly population of all ages in Shanghai from 2021 to 2030, as shown in Table 11.

**Table 11:** Basic information of the age structure of the elderly population in Shanghai from 2021 to 2030 (unit: 10^4^ people)

Year	Total population	Age			
		**60 + **	**60–69**	**70–79**	**80 + **
2021	1482.82	549.33	308.31	162.72	83.50
2022	1487.51	565.14	316.54	173.19	84.20
2023	1492.17	580.92	324.51	185.46	84.81
2024	1496.80	596.66	332.24	198.33	85.32
2025	1501.41	612.37	339.75	211.60	85.76
2026	1506.01	628.04	347.03	223.46	86.13
2027	1510.56	643.68	354.12	235.93	86.43
2028	1515.12	659.28	361.00	246.60	86.68
2029	1519.67	674.84	367.70	258.49	86.87
2030	1524.20	690.37	374.22	270.29	87.02

**Fig. 9:**
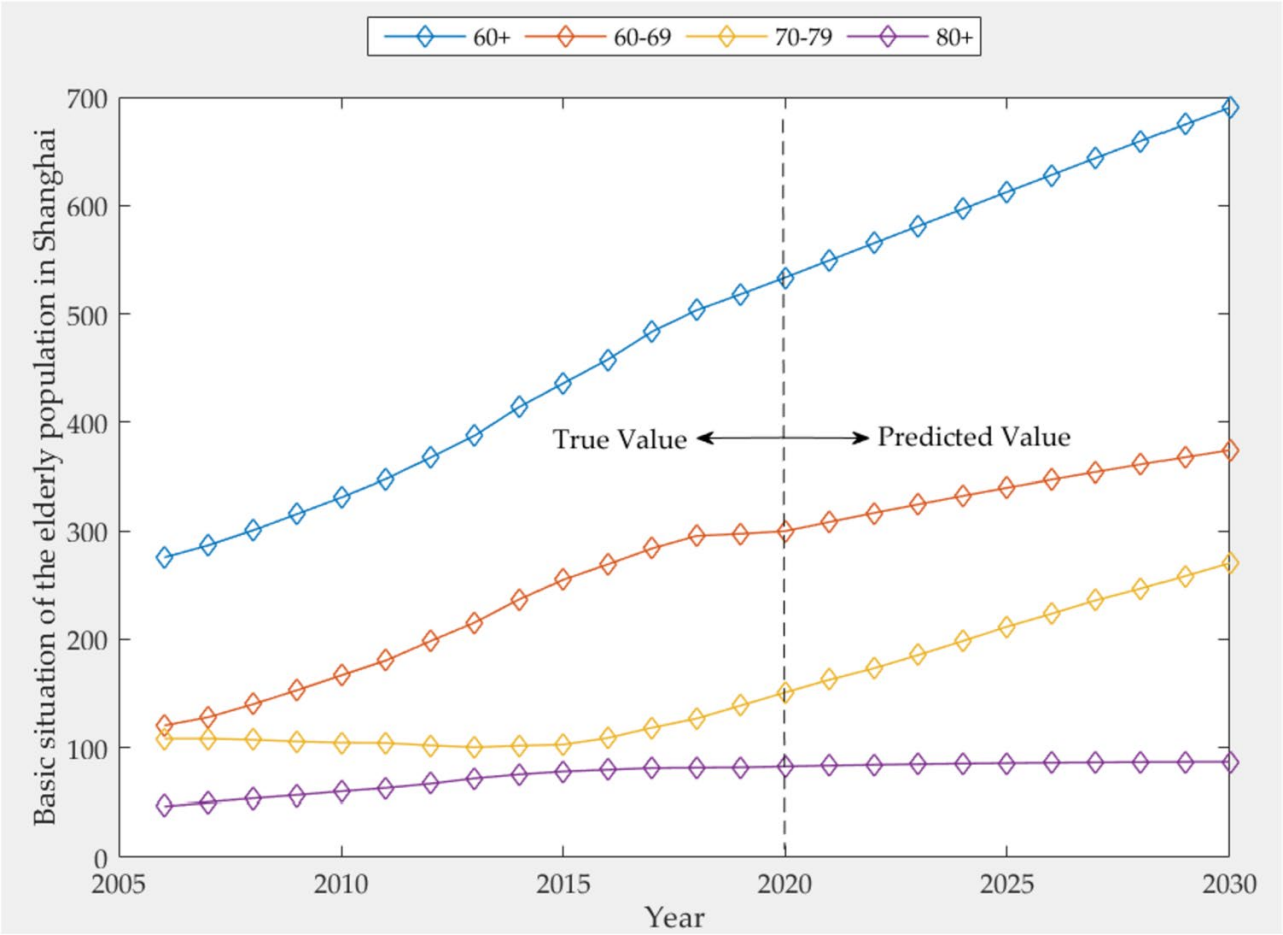
Predicted results of the elderly population in Shanghai under different age structures

As can be seen from Table 11, the number of elderly population over 60 years old has increased steadily on the whole. The elderly population aged 60–69 and 70–79 increase by 80,000 and 120,000 each year on average, with a fast growth rate, while the elderly population aged 80 and above increases by 4,000 each year, with a relatively slow growth rate. The growth trend of the age structure of the elderly population in Shanghai, as shown in Fig. 9.

## Conclusions and future work

The population problem has been affecting China's economic development, and the process of population aging is also accelerating, which is an important social problem currently facing. Accurately predicting the number of the elderly population is conducive to the formulation of relevant government policies and the positive development of the economy and society. In this paper, the GT-FGM grey prediction model is proposed, which makes use of the advantage of fractional accumulation operator that can effectively weaken the randomness of original data sequence, and combines the advantages of Theta residual optimization that can adjust the local curvature of time sequence by parameters and minimize errors to adjust parameters. The error comparison results in case analysis show that GT-FGM model has a superior prediction effect in population prediction.

### Conclusions and suggestions

The prediction results of Shanghai show that the number of elderly population in each district of Shanghai presents a trend of steady growth and obvious aging trend during 2020–2030. As the scale of the elderly population is constantly expanding, the degree of population aging is further deepening. Compared with traditional prediction models such as FGM model and GM(1,1) model, the prediction error of GT-FGM model is smaller, and its prediction accuracy is better than other prediction models. By 2030, the total elderly population in Shanghai will reach 15.2420 million, and the elderly population density in nine districts will exceed 0.5, among which Huangpu District is the highest, reaching 0.6825. The number of elderly people over the age of 60 has been steadily increasing, with the number of people aged 60 to 69, 70 to 79, 80 and above increasing by an average of 80,000, 120,000 and 4,000 each year. The difference of density distribution between regions is great, which is not conducive to the development of economy and society.

According to the prediction results, the following suggestions are present:

1. Improving the pension security system. It is suggested to increase the investment in the construction of pension infrastructure, so as to alleviate the social pressure brought by the aging population, and provide the elderly with pension insurance, medical insurance and other pension security systems to meet the needs of the elderly.
2. Reforming the medical and health care system. It is suggested to optimize the allocation of endowment and medical resources, improve the social medical security system, make medical treatment more convenient for the elderly, reform the medical security system, so that the elderly can fully enjoy the benefits of improved medical conditions.
3. Promoting balanced development among regions. According to the degree of population aging in different regions, it is suggested to formulate reasonable population policies to optimize the population age structure.
4. Developing the aging industry. It is suggested to adjust the pension policy in time according to the actual situation, develop the pension market, promote the development of the pension industry, better serve the elderly and meet their life needs.
5. Delaying the retirement age. It is suggested to reform the retirement system, raise the statutory retirement age, ease the social pressure brought about by an aging population, and allow the elderly to continue to play their role in work. This model can be applied to the research of gerontology, to predict the number and density distribution of the elderly population, and to analyze the development trend of population aging.

### Future work

Since the prediction algorithm of univariate time series is established in this paper, the relevant influencing factors are not used for prediction analysis. The main reasons include the following:

1. The development of population data is restricted by birth rate, socio-economic development, family planning policy, two-child policy and other factors, and its data characteristics have not been fully explained. It is difficult to consider all influencing factors in the model, and the complexity and diversity of population behavior make relevant factors unable to be accurately captured or explained.
2. Population aging tends to show an accelerated growth trend, and the historical statistical data information value is low, and the reliability of the model is poor. Therefore, when the actual situation changes, the prediction results of the model may lose accuracy. In addition, the structure of the model itself may lead to inflexibility of the forecast results, even when the variable factors are taken into account.
3. Currently available population data information is limited, and there is great uncertainty in the future population development. Problems such as incompatibility between different data sources and lack of temporal and spatial coverage of data will affect data quality, thus affecting the prediction results of the model.
4. If a multivariate model is constructed considering a variety of influencing factors, it usually needs to be built on some theoretical framework, but the theory itself may have defects. In addition, the selection of model structure, the setting of variables and other factors may lead to the limitations of model prediction. For example, some important variables are ignored during model construction, which may lead to inaccurate model prediction results.

Therefore, it is necessary to comprehensively consider the limitations and reliability of the model in the aspects of model establishment, parameter selection, data source and so on when making prediction analysis of population problems. At the same time, combined with the actual situation and other methods to comprehensively analyze the population problem. In the future, how to make full use of the family planning policy, two-child policy and other influencing factors, consider the use of multivariate model to fit and predict the problem of population aging, this issue will become the direction of further research and in-depth thinking.

## Data Availability

The datasets used and analyzed during the current study are available in http://shyl.mzj.sh.gov.cn/elderly_care_policy.
